# Cost-Effectiveness of α_2_ Agonists for Intravenous Sedation in Patients With Critical Illness

**DOI:** 10.1001/jamanetworkopen.2025.17533

**Published:** 2025-05-19

**Authors:** Stephen Morris, Nazir I. Lone, Cathrine A. McKenzie, Christopher J. Weir, Timothy S. Walsh

**Affiliations:** 1 Primary Care Unit, Department of Public Health and Primary Care, University of Cambridge, Cambridge, United Kingdom; 2 Usher Institute, The University of Edinburgh, Edinburgh, United Kingdom; 3 National Institute of Health and Social Care Research, Biomedical Research Centre, Southampton, Perioperative and Critical Care theme, University of Southampton, Southampton, United Kingdom

## Abstract

**Importance:**

Propofol and the α_2_ agonists dexmedetomidine and clonidine are used for sedation in patients with critical illness receiving mechanical ventilation. Evidence about the cost-effectiveness of intravenous (IV) sedation with these medications is lacking.

**Objective:**

To investigate the cost-effectiveness of dexmedetomidine-, clonidine-, and propofol-based IV sedation in patients with critical illness receiving mechanical ventilation.

**Design, Setting, and Participants:**

This economic evaluation used within-trial cost-utility analysis with a 6-month time horizon comparing dexmedetomidine-, clonidine-, and propofol-based IV sedation from a UK National Health Service and Personal Social Services perspective, with individual-level data collected from the Alpha 2 Agonists for Sedation to Produce Better Outcomes From Critical Illness (A2B) trial. Adults with critical illness receiving mechanical ventilation, with an anticipated total requirement for mechanical ventilation of at least 2 days, from 41 intensive care units in the UK were included. Recruitment ran from December 2018 through October 2023; the last date of follow-up was December 10, 2023.

**Interventions:**

Dexmedetomidine, clonidine, or propofol IV sedation. Patients receiving α_2_ agonists were permitted to receive supplemental propofol to achieve the target sedation score if required.

**Main Outcomes and Measures:**

Incremental costs and quality-adjusted life years (QALYs) gained between dexmedetomidine-based vs propofol-based and clonidine-based vs propofol-based IV sedation were assessed. Mean net monetary benefits with each medication were assessed.

**Results:**

Among 1404 adults with critical illness receiving mechanical ventilation (mean [SD] age, 59.2 [14.9] years; 901 male [64.2%]), the mean (SD) Acute Physiology and Chronic Health Evaluation (APACHE) II score was 20.3 (8.2). The incremental cost for dexmedetomidine vs propofol was $1273 (95% CI, −$5000 to $7545), and for clonidine vs propofol, it was −$1328 (−$7114 to $4459). For dexmedetomidine vs propofol, there were 0.0008 QALYs (95% CI, −0.0198 to 0.0214 QALYs) gained, and for clonidine vs propofol, there were −0.0019 QALYs (95% CI, −0.0221 to 0.0181 QALYs) gained. Mean net monetary benefits for dexmedetomidine, clonidine, and propofol were −$53 278 (95% CI, −$58 063 to −$48 493), −$50 882 (95% CI, −$55 003 to −$46 762), and −$52 036 (95% CI, −$56 230 to −$47 834), respectively, at a maximum willingness to pay for a QALY of $16 250.

**Conclusions and Relevance:**

In this study, dexmedetomidine-, clonidine-, and propofol-based IV sedation in patients with critical illness receiving mechanical ventilation had similar costs and QALYs. These findings suggest that economic considerations should not affect which sedative these patients receive.

## Key Points

**Question:**

What is the cost-effectiveness of dexmedetomidine, clonidine, and propofol for intravenous sedation in patients with critical illness receiving mechanical ventilation?

**Findings:**

In this economic evaluation using within-trial cost-utility analysis among 1404 adults with critical illness receiving mechanical ventilation, incremental costs comparing dexmedetomidine vs propofol groups and clonidine vs propofol groups were not significantly different from 0, and there were no significant differences in net monetary benefits associated with any option.

**Meaning:**

These findings suggest that economic considerations should not affect which IV sedative agent patients with critical illness receiving mechanical ventilation should receive.

## Teaser

This economic evaluation investigates the cost-effectiveness of intravenous (IV) sedation using dexmedetomidine, clonidine, or propofol in patients with critical illness receiving mechanical ventilation.

## Introduction

Most patients with critical illness receiving mechanical ventilation (MV) require sedation. Propofol is the most widely used first-line sedative medication for patients in the intensive care unit (ICU), but the α_2_ agonist dexmedetomidine is also widely used. Clonidine is an α_2_ agonist with lower α_2_-receptor selectivity, but it is used for sedation in the ICU, mostly in the UK.^1^ Despite previous research, there is uncertainty whether dexmedetomidine-based sedation is clinically superior to propofol-based sedation, and safety concerns remain about the use of dexmedetomidine. To our knowledge, the safety and effectiveness of clonidine-based sedation have not been studied in large randomized clinical trials. Systematic reviews and meta-analyses of dexmedetomidine suggest that it may be associated with reduced delirium, duration of MV, and ICU length of stay, with no overall associations with mortality.^2,3^ The largest randomized clinical trial found no effect on mortality but suggested possible heterogeneity of treatment effects by age, with younger patients experiencing increased mortality and older patients experiencing decreased mortality compared with usual care with propofol, benzodiazepines, or both.^4,5,6^

The Alpha 2 Agonists for Sedation to Produce Better Outcomes From Critical Illness (A2B) trial was a 3-armed randomized clinical trial comparing dexmedetomidine-, clonidine-, and propofol-based primary sedation for patients with critical illness receiving MV.^7^ The study found that neither dexmedetomidine- nor clonidine-based IV sedation was superior to propofol-based IV sedation in reducing time to successful extubation.^8^

To our knowledge, there have been 3 economic analyses conducted of dexmedetomidine for sedation in adult patients in the ICU receiving MV.^9,10,11^ All concluded that use of dexmedetomidine may be associated with cost savings, but these studies were conducted before recent issues about the safety of dexmedetomidine were published, and none included clonidine. In addition, dexmedetomidine is now available off patent at substantially lower cost than Dexdor (UK licensed brand; Orion Pharma) and Precedex (US licensed brand; Pfizer). We therefore undertook an economic evaluation to investigate the cost-effectiveness of dexmedetomidine-, clonidine-, and propofol-based IV sedation using A2B trial data.

## Methods

Methods for this economic evaluation were predefined in a health economic analysis plan (eAppendix 1 in Supplement 1). The study is reported following the Consolidated Health Economic Evaluation Reporting Standards (CHEERS) 2022 reporting guideline. Ethical approval for the A2B trial was obtained from the Scotland A Research Ethics Committee. Signed consent in the A2B trial was obtained after consultation with surrogate decision-makers, with deferred consent if these were unavailable within 2 hours of confirming eligibility. When deferred consent was used, consent from the surrogate decision-maker was sought at the earliest opportunity. All patients were approached if they regained capacity to provide consent to remain in the trial. The ethical approval and consenting process from the A2B trial included this study. Further detail is provided in the main trial publication.^8^

### Trial Background and Summary of Main Results

A2B was an open-label, 3-arm trial randomizing 1437 adults with critical illness within 48 hours of starting MV expected to require 48 or more hours of further MV to receive dexmedetomidine-, clonidine-, or propofol-based sedation.^7^ The trial took place in 41 ICUs in the UK. Recruitment ran from December 2018 through October 2023, and the last date of follow-up was December 10, 2023. Previous patients were involved in trial outcome choice and assisted with trial conduct.^7^ The sedation target was a Richmond Agitation and Sedation Scale score of −2 to 1 (a calm and cooperative patient) unless deep sedation was clinically indicated. Patients receiving α_2_ agonists were allowed to receive supplemental propofol to achieve target sedation if required. The primary outcome was time from randomization to successful extubation, defined as extubation followed by 48 hours of spontaneous breathing without MV. Median times to extubation were similar for propofol (162 hours [95% CI, 136-170 hours]), dexmedetomidine (136 hours [95% CI, 117-150 hours]), and clonidine (146 hours [95% CI, 124-168 hours]). Hazard ratios for time to successful extubation were 1.09 (95% CI, 0.96-1.25; *P* = .20) for dexmedetomidine vs propofol and 1.05 (95% CI, 0.95-1.17; *P* = .34) for clonidine vs propofol.^8^ Among secondary outcomes, agitation occurred more frequently with both α_2_ agonists and delirium rates were similar over the 3 arms. Rates of severe bradycardia were 60% higher with both α_2_ agonists compared with propofol. There were no differences in time to ICU discharge among surviving patients. Median ICU stays after randomization were 12 days (95% CI, 11-13 days) for propofol, 11 days (95% CI, 10-12 days) for dexmedetomidine, and 12 days (95% CI, 10-13 days) for clonidine.^8^ A detailed description of the sedation practice in each trial group is included in the main trial report.^8^ Briefly, patients in the propofol group received propofol for a median (IQR) 4 (2-8) days after randomization, and during days 2 to 7 after randomization, the median daily propofol dose was 22 to 26 mg/kg/d. In the dexmedetomidine group, patients received dexmedetomidine for a median (IQR) 4 (2-7) days after randomization at a median dose ranging from 9 to 15 µg/kg/24 hours over days 2 to 7. Patients also received propofol on 77% of days; the median daily dose ranged from 4 to 7 mg/kg/24 hours. In the clonidine group, patients received clonidine for a median (IQR) 4 (2-7) days after randomization at a median dose ranging from 15 to 22 µg/kg/24 hours over days 2 to 7. Patients also received propofol on 76% of days; the median daily dose ranged from 8 to 10 mg/kg/24 hours. Use of additional benzodiazepines as adjunct or rescue sedatives was very low. The main conclusion of the trial was that in patients with critical illness receiving MV, neither dexmedetomidine- nor clonidine-based IV sedation was superior to propofol-based sedation in reducing time to successful extubation.^8^

### Overview of Economic Evaluation

We undertook a cost-utility analysis to compare dexmedetomidine-, clonidine-, and propofol-based IV sedation from a UK National Health Service (NHS) and Personal Social Services perspective using A2B trial data. The analysis was based on patient-level resource use, mortality, and health-related quality of life data assessed in the trial between baseline and 6-month follow-up. The outcome measure was quality-adjusted life years (QALYs).^12^ Cost-effectiveness was expressed in terms of net monetary benefits (NMBs).^12^ The time horizon was 6 months, reflecting the follow-up period in the trial. Extrapolation beyond the end of the trial was not undertaken because there was no evidence of differences in costs or benefits between groups at 6 months. Given the time horizon, discounting was not applied. All costs were calculated in 2023 to 2024 UK pounds sterling and converted to and presented in US dollars (UK £1 = $1.25).^13^

### Resource Use and Costs

For every patient, we calculated the cost of index hospitalization from ICU admission to hospital discharge and at 6 months postrandomization follow-up based on resource use data collected in the trial. We included costs of dexmedetomidine, clonidine, propofol, other IV opioids, sedatives as rescue medications, antipsychotic medications, and length of stay in the hospital in ICU and regular inpatient wards collected using case report forms. Postdischarge costs included general practitioner (GP) contacts; nurse contacts; NHS physiotherapist, occupational therapist, speech therapist, and dietitian contacts; home care worker and social worker contacts; psychological therapist and counselor contacts; day hospital contacts; aides and adaptation worker contacts; substance misuse nurse contacts; Macmillan nurse contacts; accident and emergency department visits; outpatient visits; hospital readmissions; rehabilitation hospital admissions; and care home admissions. These resource use data were collected using patient questionnaires (eAppendix 2 in Supplement 1).

Unit costs were from published sources^14,15,16^ inflated to 2023 to 2024 values using NHS Pay and Prices Indices^14^ and converted to US dollars. Costs for medications were based on quantities recorded up to the achievement of the primary outcome (successful extubation) truncated at day 28 after admission to the ICU. Unit costs for the 3 study drugs were based on December 2023 figures (dexmedetomidine: 400 μg/4 mL solution for infusion vials, pack size 10 = $48.68; clonidine: 150 µg single ampoule = $0.75; propofol: 200 mg/20mL emulsion for injection ampoules, pack size 5 = $3.49). Alternative values were used in sensitivity analysis, described subsequently. In the UK, ICU costs per day are available based on the number of organs supported, so ICU costs were based on this number, up to a maximum of 4 organs, recorded daily for every patient in the trial until the achievement of the primary outcome, allowing for changes in intensity of care over time. Types of organ support identified were respiratory support (defined by receipt of MV), cardiovascular support (receipt of inotropic agents or vasopressors), kidney support (receipt of kidney replacement therapy), and liver support (liver Sequential Organ Failure Assessment [SOFA] score of 3 or 4). After the primary outcome was achieved, no data were collected on the number of organs supported, so this was assumed to be 0 organs. Given that this was likely to underestimate ICU costs, daily ICU costs were varied in sensitivity analysis. Unit costs of regular hospital inpatient ward stays were daily costs applied to the length of stay. Unit costs for postdischarge resource use were based on costs per contact or visit.

### Utilities and QALYs

QALYs were estimated using utility scores with the 5-level EQ-5D (EQ-5D-5L) instrument^17^ collected at 30 days and 3 and 6 months. Given that patients recruited to the trial were critically ill, completion of the EQ-5D-5L at baseline was not possible. In the base case, we calculated QALYs assuming a baseline utility score of 0.^18,19^ Participants were asked to retrospectively record their baseline EQ-5D-5L score at 30 days of follow-up, and baseline EQ-5D-5L scores for participants were also recorded by proxy respondents; both scores were used in sensitivity analysis. All EQ-5D-5L data were assigned to baseline, 30 days, and 3- or 6-month measurement points, irrespective of precisely when they were measured. EQ-5D-5L data were converted to utility scores using recommended preference weights at the time of analysis.^20^ Patients who died were assigned a utility value of 0 at the date of death and all subsequent time periods. Patient-specific utility profiles were constructed assuming a straight-line relation between utility scores at each follow-up point. QALYs experienced by each patient were calculated as the area underneath this profile.

### Missing Data

Missing data across individual variables ranged from 1% to 53%, with missing data most prevalent for utility scores. We assumed these data were missing at random, and multiple imputation was used to impute missing data for utility scores, study drug costs, concomitant medications, ICU days, regular inpatient ward days, and postdischarge costs. Age, sex, study site, and whether the patient had died at 30 days, 3 months, or 6 months were included in the imputation as additional explanatory variables. We used an iterative Markov chain Monte Carlo procedure based on multivariate normal regression and generated 20 imputed datasets.

### Statistical Analysis

Analyses were performed on an intention-to-treat basis. Raw mean (SD) resource use, total costs, utility scores, and QALYs per participant were estimated for each randomized group; given that these were raw values, they were unadjusted, with no imputation for missing data. After multiple imputation, we calculated differences in mean costs and QALYs of dexmedetomidine vs propofol groups and clonidine vs propofol groups using regression analysis adjusting for study site as a fixed effect. NMBs for patients allocated to receive sedation based on dexmedetomidine (*d*), clonidine (*c*), or propofol (*p*) were calculated as the mean QALYs per patient (*QALY*) multiplied by the maximum willingness to pay for a QALY (*R*) minus the mean cost per patient (*COST*):

*NMB_i_* = *QALY_i_* × *R* − *COST_i_* for *i* = *d*,*c*,*p*

We used UK cost-effectiveness thresholds of £13 000 ($16 250), £20 000 ($25 000), and £30 000 ($37 500) as willingness-to-pay values for a QALY (*R*).^21,22^ NMBs were likely to be negative, reflecting a relatively high cost during the first 6 months and the likelihood of limited QALYs being accrued. The treatment option with the highest NMB (most positive/least negative value) is preferred on cost-effectiveness grounds. For each of 20 imputed datasets, we ran 1000 bootstrap replications and calculated standard errors around mean values accounting for uncertainty in imputed values, skewed cost data and utility values, and sampling variation.^23^ Standard errors were used to calculate 95% CIs. All analyses were performed using Stata statistical software version 15.1 (StataCorp).^24^

We conducted sensitivity and subgroup analyses. Cost-effectiveness acceptability curves^25^ showing the probability that each option was cost-effective at different values for the maximum willingness to pay for a QALY were generated based on the proportion of bootstrap replications across all 20 imputed datasets where NMBs for each option were highest. We report the probability that each option was cost-effective at a maximum willingness to pay for a QALY of $16 250, $25 000, and $37 500. We undertook deterministic sensitivity analyses: (1) complete case analysis without imputing missing values; (2) baseline utility scores based on retrospectively scored values by patients; (3) baseline utility scores based on proxy respondent values; (4) ICU cost per day calculated as the national mean cost per day for each number of organs supported (0, 1, 2, 3, 4, 5, and ≥6) weighted by the national mean distribution of the number of organs supported each day ($2913/d); and (5) unit costs of study drugs based on daily UK costs for sedating a 70-kg adult receiving MV at 10 A2B sites (dexmedetomidine, $28; clonidine, $10; propofol, $19), with study drug costs calculated by multiplying these by the number of days receiving each medication. In predefined subgroup analyses undertaken in the main trial, we investigated cost-effectiveness by baseline age 64 years or older vs younger than 64 years; less than vs greater than the median baseline Prediction of Delirium in ICU Patients (PRE-DELIRIC^26^) delirium risk prediction score; less than vs greater than the median baseline SOFA score; and with vs without sepsis at enrollment.

## Results

### Descriptive Statistics

Among 1404 participants in the analysis population (mean [SD] age, 59.2 [14.9] years; 901 male [64.2%]), the mean (SD) Acute Physiology and Chronic Health Evaluation (APACHE) II score was 20.3 (8.2). Baseline patient characteristics were well-balanced between study groups.^8^ The mean (SD) number of days that patients in the dexmedetomidine group received dexmedetomidine, clonidine, and propofol was 6.5 (5.4) days, 0.2 (0.9) days, and 5.8 (5.4) days, respectively (Table 1). For the clonidine group, the figures were 0.2 (1.1) days, 6.3 (5.3) days, and 5.9 (5.1) days, respectively. For the propofol group, the numbers were 0.4 (1.7) days, 1.1 (3.0) days, and 6.7 (5.3) days, respectively. The mean (SD) combined cost per participant of all 3 drugs up to 28 days was $169 ($203) for the dexmedetomidine group, $116 ($135) for the clonidine group, and $81 ($111) for the propofol group. Combined daily costs in each group decreased over time (eFigure in Supplement 1). The mean (SD) combined cost per participant of the included concomitant medications was $68 ($104) for the dexmedetomidine group, $70 ($101) for the clonidine group, and $71 ($114) for the propofol group (Table 1).

**Table 1.** Resource Use and Costs

Outcome	Mean (SD)			Unit costs, $^a^
	Dexmedetomidine (n = 449)	Clonidine (n = 468)	Propofol (n = 470)	
**Resource use and costs during index hospitalization**				
Days receiving study drug				
Dexmedetomidine	6.5 (5.4)	0.2 (1.1)	0.4 (1.7)	NA
Clonidine	0.2 (0.9)	6.3 (5.3)	1.1 (3.0)	NA
Propofol	5.8 (5.4)	5.9 (5.1)	6.7 (5.3)	NA
Costs of study drugs, $^a^				
Dexmedetomidine	123 (152)	4 (30)	8 (40)	NA^b^
Clonidine	1 (9)	68 980)	11 (39)	NA^b^
Propofol	44 (68)	45 (63)	63 (79)	NA^b^
Combined	169 (203)	116 (135)	81 (111)	NA
Costs of concomitant medications, $^a^				
IV opioids	65 (100)	67 (96)	67 9104)	NA^c^
Sedatives (rescue medications)	2 (13)	4 (20)	5 (30)	NA^c^
Antipsychotics	0 (2)	0 (3)	0 (2)	NA^c^
Combined	68 (104)	70 (101)	71 (114)	NA
Length of hospitalization, d				
ICU^d^				
Total	15.6 (20.5)	14.9 (16.3)	14.7 (15.9)	
By No. of organs supported				
0	7 (16.6)	6.1 (11.5)	5.8 (11.5)	1981
1	3.4 (4.4)	3.4 (4.6)	3.7(5.4)	2258
2	3.9 (4.2)	4.1 (4.0)	4.0 (4.0)	3025
3	1.2 (2.8)	1.1 (2.6)	1.3(2.7)	3389
4	0.1 (0.8)	0.1(0.8)	0.1 (0.7)	3671
Regular ward	17.7 (28.2)	15.8 (24.0)	17.9 (28.3)	823
Total hospital	33.3 (36.0)	30.7 (31.0)	32.8 (34.7)	NA
Costs of hospitalization, $^a^^,^^e^				
Total ICU	38 095 (44 519)	36 865 (36 740)	36 598 (35 717)	NA
Regular ward	14 579 (23 211)	12 991 (19 735)	14 711 (23 311)	NA
Total hospital	52 884 (51 761)	49 825 (43 720)	51 781 (45 448)	NA
**Resource use and costs from hospital discharge to 90 d^f^**				
Resource use				
Participants, No.	240	263	276	NA
GP contacts at the GP surgery	0.4 (1.2)	0.4 (0.9)	0.4 (1.2)	61
GP contacts at home	0.1 (0.4)	0.0 (0.4)	0.1 (0.5)	123
GP contacts by telephone	0.4 (1.1)	0.6 (1.5)	0.5 (1.2)	11
District nurse contacts	1.2 (5.7)	1.5 (7.5)	1.4 (6.9)	46
Practice nurse contacts	0.6 (3.2)	0.5 (2.4)	0.5 (4.0)	23
NHS physiotherapist contacts	0.2 (0.9)	0.8 (4.6)	0.3 (1.5)	89
Occupational therapist contacts	0.1 (0.6)	0.2 (1.4)	0.2 (1.3)	70
Speech therapist contacts	0.0 (0.1)	0.0 (0.2)	0.0 (0.1)	140
Dietitian contacts	0.0 (0.2)	0.2 (1.4)	0.1 (0.5)	104
Home care worker contacts	0.2 (1.6)	0.6 (5.2)	0.1 (2.1)	34
Social worker contacts	0.0 (0.1)	0.0 (0.2)	0.1 (1.3)	66
Psychological therapist contacts	0.1 (1.0)	0.0 (0.2)	0.1 (0.3)	70
Counselor contacts	0.1 (0.4)	0.0 (0.1)	0.0 (0.3)	70
Day hospital contacts	0.1 (0.7)	0.2 (1.1)	0.2 (1.1)	133
Aides and adaptation worker contacts	0.0 (0.3)	0.1 (0.5)	0.1 (0.4)	70
Substance misuse nurse contacts	0.0 (0.3)	0.0 (0.2)	0.0 (0.0)	116
Macmillan nurse contacts	0.0 (0.1)	0.0 (0.7)	0.0 (0.1)	46
Accident and emergency department visits	0.1 (0.4)	0.1 (0.4)	0.1 (0.5)	303
Outpatient visits	0.7 (2.5)	0.6 (1.7)	0.7 (1.9)	271
Readmissions to hospital	0.1 (0.2)	0.1 (0.3)	0.1 (0.2)	5899
Admissions to rehabilitation	0.0 (0.2)	0.0 (0.2)	0.0 (0.2)	1071
Care home admissions	0.0 (0.1)	0.0 (0.1)	0.0 (0.1)	2078
Costs for all contacts, $^a^	845 2206	1155 (3073)	954 (2516)	
**Resource use and costs from 9-180 d^f^**				
Resource use				
Participants, No.	212	232	240	NA
GP contacts at GP surgery	0.5 (1.4)	0.3 (0.9)	0.6 (1.6)	61
GP contacts at home	0.2 (1.8)	0.1 (0.7)	0.1 (0.3)	123
GP contacts by telephone	0.6 (1.8)	0.6 (1.4)	0.7 (1.7)	11
District nurse contacts	0.4 (2.4)	0.9 (4.7)	1.5 (9.0)	46
Practice nurse contacts	0.6 (3.0)	0.5 (2.6)	0.7 (5.5)	23
NHS physiotherapist contacts	0.4 (2.1)	0.4 (2.0)	0.3 (1.8)	89
Occupational therapist contacts	0.1 (1.4)	0.1 (0.4)	0.1 (0.5)	70
Speech therapist contacts	0.0 (0.1)	0.1 (0.4)	0.1 (0.4)	140
Dietitian contacts	0.1 (0.3)	0.1 (0.4)	0.1 (0.3)	104
Home care worker contacts	0.2 (2.6)	0.2 (3.2)	0.0 (0.0)	34
Social worker contacts	0.0 (0.2)	0.0 (0.3)	0.0 (0.1)	66
Psychological therapist contacts	0.1 (0.4)	0.1 (1.1)	0.2 (0.8)	70
Counselor contacts	0.1 (0.6)	0.0 (0.7)	0.1 (0.7)	70
Day hospital contacts	0.3 (1.4)	0.3 (1.1)	0.1 (0.7)	133
Aides and adaptation worker contacts	0.0 (0.2)	0.1 (0.7)	0.2 (1.4)	70
Substance misuse nurse contacts	0.0 (0.3)	0.0 (0.4)	0.0 (0.1)	116
Macmillan nurse contacts	0.1 (0.9)	0.1 (0.8)	0.0 (0.1)	46
Accident and emergency department visits	0.1 (0.4)	0.1 (0.3)	0.1 (0.6)	303
Outpatient visits	0.6 (1.6)	0.7 (1.9)	0.6 (1.4)	271
Readmissions to hospital	0.0 (0.2)	0.0 (0.2)	0.0 (0.2)	5899
Admissions to rehab	0.0 (0.1)	0.0 (0.1)	0.0 (0.1)	1071
Care home admissions	0.0 (0.0)	0.0 (0.1)	0.0 (0.1)	2078
Costs for all contacts, $^a^	693 (1910)	750 (2367)	853 (3004)	
**Costs from hospital discharge to 180 d**				
Participants, No.	186	214	217	NA
Costs for all contacts, $^a^	1034 (3066)	1718 (4995)	1498 (4663)	NA
**Total costs**				
Participants, No.	186	214	217	NA
Total costs, $^a^	55 423 (61 193)	49 091 (44 743)	51 566 (49 226)	NA

Abbreviations: GP, general practitioner; ICU, intensive care unit; IV, intravenous; NA, not applicable; NHS, National Health Service.

^a^ Costs are in 2023 to 2024 US dollars (UK £1 = $1.25).^13^

^b^ Unit costs were determined for dexmedetomidine (400 µg/4 mL solution for infusion vials, pack size 10 = $48.68), clonidine (150 µg ampoule = $0.75), and propofol (200 mg/20 mL emulsion for injection ampoules, pack size 5 = $3.49).

^c^ IV opioids included fentanyl, alfentanil, morphine, and remifentanil. Sedatives used as rescue medications included midazolam and diazepam. Antipsychotics included haloperidol. Unit costs were determined for fentanyl (500 µg/10 mL solution for injection ampoules, pack size 10 = $5.29), alfentanil (5 mg/10 mL solution for injection ampoules, pack size 10 = $17.68), morphine (10 mg/1 mL solution for injection ampoules, pack size 10 = $2.38), remifentanil (5 mg powder for solution for injection vials, pack size 5 = $21.48), midazolam (10 mg/2 mL solution for injection ampoules, pack size 10 = $3.85), diazepam (10 mg/2 mL solution for injection ampoules, pack size 10 = $11.11), and haloperidol (5 mg/1 mL solution for injection ampoules, pack size 10 = $29.84).

^d^ ICU costs were based on the number of organs supported, up to a maximum of 4 organs, recorded daily for every patient in the trial until the achievement of the primary outcome. After the achievement of the primary outcome, no data were collected on the number of organs supported, so this was assumed to be 0.

^e^ Unit costs are per day.

^f^ Unit costs are per contact or visit.

The mean (SD) total number of ICU days per participant was 15.6 (20.5) days in the dexmedetomidine group, 14.9 (16.3) days in the clonidine group, and 14.7 (15.9) days in the propofol group (Table 1). The mean (SD) total length of stay in the hospital was 33.3 (36.0) days, 30.7 (31.0) days, and 32.8 (34.7) days in dexmedetomidine, clonidine, and propofol groups, respectively. Mean (SD) total ICU costs per participant in each group were $38 095 ($44 519), $36 865 ($36 740), and $36 598 ($35 717) for dexmedetomidine, clonidine, and propofol groups, respectively, and mean (SD) total hospital costs were $52 884 ($51 761), $49 825 ($43 720), and $51 781 ($45 448]) for dexmedetomidine, clonidine, and propofol groups, respectively. In each group, the combined cost of study drugs accounted for less than 1% of total ICU costs.

After discharge, surviving patients in all 3 groups used primary and community care services up to 180 days (Table 1). The most common contacts were GP contacts, district and practice nurse contacts, and outpatient visits. The mean (SD) cost per participant from hospital discharge to 180 days was $1034 ($2066) in the dexmedetomidine group, $1718 ($4995) in the clonidine group, and $1498 ($4663) in the propofol group. Total costs from hospital discharge to 180 days were 10% to 15% of hospitalization costs. Mean (SD) total costs per participant were $55 423 ($61 193) in the dexmedetomidine group, $49 091 ($44 743) in the clonidine group, and $51 566 ($49 226]) in the propofol group.

Mean utility values per participant were similar for the 3 groups and decreased over time (Table 2). In the base case (assuming a baseline utility score of 0), mean (SD) total QALYs per participant up to 6 months were 0.08 (0.14) in the dexmedetomidine group, 0.08 (0.13) in the clonidine group, and 0.08 (0.14) in the propofol group. Baseline utility scores that were retrospectively scored or based on proxy responses were higher than the base case assumption.

**Table 2.** EQ-5D-5L Utility Scores and QALYs

Outcome	Dexmedetomidine		Clonidine		Propofol	
	Participants, No.	Mean (SD)	Participants, no.	Mean (SD)	Participants, No.	Mean (SD)
EQ-5D-5L utility score						
Baseline scored retrospectively	171	0.73 (0.31)	174	0.73 (0.30)	190	0.71 (0.30)
Baseline by proxy	121	0.63 (0.33)	105	0.60 (0.34)	96	0.55 (0.37)
At 30 d	301	0.28 (0.37)	313	0.26 (0.35)	329	0.28 (0.36)
At 90 d	214	0.25 (0.35)	241	0.24 (0.33)	253	0.25 (0.35)
At 180 d	201	0.21 (0.35)	228	0.22 (0.34)	229	0.21 (0.34)
QALYs						
Baseline = 0	152	0.08 (0.14)	181	0.08 (0.13)	175	0.08 (0.14)
Baseline scored retrospectively	41	0.31 (0.12)	61	0.27 (0.12)	51	0.24 (0.16)
Baseline scored by proxy	49	0.15 (0.16)	50	0.15 (0.16)	45	0.12 (0.14)

Abbreviations: EQ-5D-5L, 5-level EQ-5D; QALY, quality-adjusted life year.

### Main Outcomes

Neither incremental costs nor QALYs gained comparing dexmedetomidine vs propofol groups or clonidine vs propofol groups were significantly different from 0 (Table 3). Incremental costs for dexmedetomidine vs propofol were $1273 (95% CI, −$5000 to $7545). For clonidine vs propofol, they were −$1328 (95% CI, −$7114 to $4459). For dexmedetomidine vs propofol, there were 0.0008 QALYs (95% CI, −0.0198 to 0.0214 QALYs) gained. For clonidine vs propofol, there were −0.0019 QALYs (95% CI, −0.0221 to 0.0181 QALYs) gained.

**Table 3.** Incremental Costs and QALYs Gained

Analysis	Mean (95% CI)	
	Incremental cost, $^a^	QALYs gained
**Whole sample**		
Base case, multiple imputation with adjustment		
Dexmedetomidine vs propofol	1273 (−5000 to 7545)	0.0008 (−0.0198 to 0.0214)
Clonidine vs propofol	−1328 (−7114 to 4459)	−0.0019 (−0.0221 to 0.0181)
**Sensitivity analyses**		
Complete case analysis with adjustment		
Dexmedetomidine vs propofol	3864 (−6978 14704)	0.0086 (−0.0216 to 0.0388)
Clonidine vs propofol	−1875 (−10801 to 7063)	0.0080 (−0.0201 to 0.0361)
QALYs		
Baseline scored retrospectively		
Dexmedetomidine vs propofol	1273 (−5000 to 7545)	0.0018 (−0.0215 to 0.0250)
Clonidine vs propofol	−1328 (−7114 to 4459)	−0.0012 (−0.0241 to 0.0217)
Baseline scored by proxy		
Dexmedetomidine vs propofol	1273 (−5000 to 7545)	0.0015 (−0.0208 to 0.0238)
Clonidine vs propofol	−1328 (−7114 to 4459)	−0.0009 (−0.0232 to 0.0214)
ICU days, cost £2330 per day		
Dexmedetomidine vs propofol	2185 (−5695 to 10065)	0.0008 (−0.0198 to 0.0214)
Clonidine vs propofol	−1236 (−8294 to 5822)	−0.0019 (−0.0221 to 0.0181)
Study drug costs based on mean costs per day across 10 A2B sites		
Dexmedetomidine vs propofol	1202 (−5082 to 7486)	0.0008 (−0.0198 to 0.0214)
Clonidine vs propofol	−1451 (−7235 to 4333)	−0.0019 (−0.0221 to 0.0181)

Abbreviations: A2B, Alpha 2 Agonists for Sedation to Produce Better Outcomes From Critical Illness; QALY, quality-adjusted life year.

^a^ Costs are in 2023 to 2024 US dollars (UK £1 = $1.25).^13^ All analyses were undertaken using base case assumptions other than where indicated. Data include values imputed using multiple imputation (described previously) with adjustment for study site, except for the complete case analysis with adjustment, where there was no multiple imputation of missing values.

Mean NMBs for dexmedetomidine, clonidine, and propofol were −$53 278 (95% CI, −$58 063 to −$48 493), −$50 882 (95% CI, −$55 003 to −$46 762), and −$52 036 (95% CI, −$56 230 to −$47 834), respectively, at a maximum willingness to pay for a QALY of $16 250 (Table 4). Values were similar at a maximum willingness to pay for a QALY of $25 000 and $37 500.

**Table 4.** Net Monetary Benefits

Analysis	Net monetary benefits, mean (95% CI), $^a^	Probability of being cost-effective^b^
**Maximum willingness to pay for a QALY = $16 250**		
Dexmedetomidine	−53 278 (−58 063 to −48 493)	0.15
Clonidine	−50 882 (−55 003 to −46 762)	0.56
Propofol	−52 036 (−56 230 to −47 834)	0.29
**Maximum willingness to pay for a QALY = $25 000**		
Dexmedetomidine	−51 824 (−56 640 to −47 007)	0.15
Clonidine	−49 456 (−53 599 to −45 312)	0.56
Propofol	−50 603 (−54 829 to −46 377)	0.29
**Maximum willingness to pay for a QALY = $37 500**		
Dexmedetomidine	−49 747 (−54 615 to −44 879)	0.16
Clonidine	−47 418 (−51 600 to −43 235)	0.55
Propofol	−48 556 (−52 834 to −44 278)	0.29

Abbreviation: QALY, quality-adjusted life year.

^a^ Net monetary benefits are in 2023 to 2024 US dollars (UK £1 = $1.25).^13^ All analyses were undertaken using base case assumptions. Data include values imputed using multiple imputation (described previously) with adjustment for study site. The 95% CIs were derived from 1000 bootstrap replications of each of 20 imputed datasets (described previously).

^b^ The probability that each option is cost-effective at that maximum willingness to pay for a QALY.

### Sensitivity Analyses

At a maximum willingness to pay for a QALY of $16 250, the probability that dexmedetomidine-, clonidine-, and propofol-based IV sedation were preferred on cost-effectiveness grounds was 0.15, 0.56, and 0.29, respectively (Table 4; Figure). At a maximum willingness to pay for a QALY of $25 000, these values were also 0.15, 0.56, and 0.29, respectively. At a maximum willingness to pay for a QALY of $37 500, they were 0.16, 0.55, and 0.29, respectively. The more favorable values for clonidine were driven by the nonsignificant but lower total costs and therefore less negative NMBs in this group.

**Figure.**
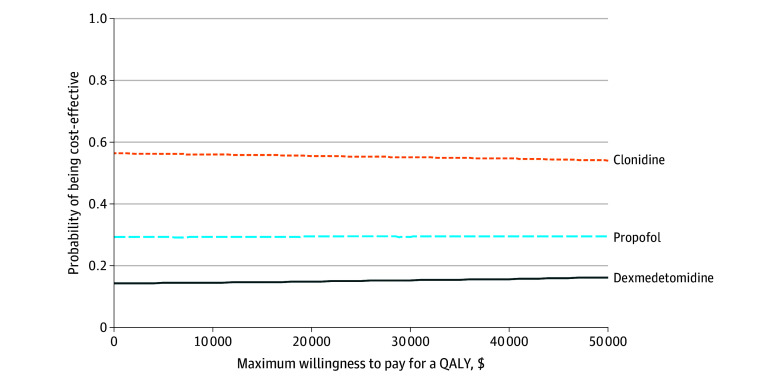
Cost-Effectiveness Acceptability Curve The figure shows the probability that each study drug is cost-effective at different values of the maximum willingness to pay for a quality-adjusted life year (QALY). Costs are in 2023 to 2024 US dollars (UK £1 = $1.25).^13^ All analyses were undertaken using base case assumptions. Data include values imputed using multiple imputation (described previously) with adjustment for study site.

In all deterministic sensitivity analyses, incremental costs and QALYs gained for dexmedetomidine vs propofol and for clonidine vs propofol remained not significantly different from 0 (Table 3). Further details of study drug costs based on costs at 10 A2B sites are in eTable 1 in Supplement 1. In all subgroups, incremental costs and QALYs gained were not significantly different from 0 (eTable 2 in Supplement 1).

## Discussion

In this economic evaluation of the A2B trial, dexmedetomidine-, clonidine-, and propofol-based sedation in patients with critical illness receiving MV had similar costs, QALYs, and NMBs from the perspective of the UK NHS. This is consistent with findings from the A2B trial, which showed that neither dexmedetomidine- nor clonidine-based sedation was superior to propofol-based sedation in reducing time to successful extubation or other important clinical outcomes. Sensitivity analyses and subgroup analyses showed little uncertainty in these findings. Our findings suggest that there is no reason to prefer dexmedetomidine-, clonidine-, or propofol-based IV sedation on economic grounds. Other factors should be taken into account when deciding which option to use, such as the balance between efficacy and harm and medicines optimization.^27^ While this study was based in the UK, the findings are likely to be relevant to other settings given that there were no between-group differences in length of stay in the trial and study drugs accounted for less than 1% of total ICU costs. This suggests that even if the relative cost of IV sedation drugs differs between countries compared with the UK, this is unlikely to affect their cost-effectiveness. Dexmedetomidine has been available from multiple suppliers off patent since 2019, and based on publicly available data, the mean wholesale price for a 1000-µg (10-mL) vial of dexmedetomidine in the US is $159.99 (April 2025). The current British National Formulary price for the same UK generic ampoule is similar, at £78.25 ($97.81).

The main strength of our analysis is that it is based on a large multicenter randomized clinical trial with detailed information on resource use, utility values, and mortality. The population studied was broad, and the design was pragmatic, increasing generalizability. We also adapted our health economic analyses to reflect changes in drugs costs during the tenure of the A2B trial; this was especially important given that dexmedetomidine became available as a generic medication during the trial period.

### Limitations

This study has several limitations. First, we undertook a within-trial analysis over a 6-month period. We could have used a longer time horizon, but there were no differences in costs or benefits between study groups at this point, so this would not have affected incremental analyses. Second, the intervention was unblinded, and the primary outcome was measured by unblinded researchers, which introduces potential bias. Third, the A2B trial was a pragmatic effectiveness trial, and we cannot exclude different results if trial interventions had been applied differently. In the trial, patients receiving α_2_ agonists were allowed to receive supplemental propofol if the maximum α_2_ agonist dose was reached or because of clinician concerns or dose-limiting adverse effects. Most patients continued to receive some propofol but at a substantially lower dose than in the usual care propofol group. Fourth, the analysis was undertaken from the perspective of the UK NHS and Personal Social Services. While this is the recommended perspective in the UK,^21^ a wider (eg, a societal) perspective would also include associated costs for the rest of society, including patients, families, and businesses. Additionally, results may not be generalizable to the US or other settings outside the UK (eg, depending on the relative value of unit costs in different countries). Fifth, we did not have complete data for every participant in the trial, especially after hospital discharge. We used multiple imputation to address this, and conclusions were similar whether we used multiple imputation or complete case analysis.

## Conclusions

In this economic evaluation using within-trial cost-utility analysis involving 1404 adults with critical illness receiving MV, incremental costs between dexmedetomidine and propofol groups and clonidine and propofol groups were not significantly different from 0, and there were no significant differences in NMBs associated with either option. These findings suggest that IV sedation selection among dexmedetomidine, propofol, and clonidine should be based on individual patient need rather than economic considerations.

## Notes

Supplement 1. **eAppendix 1.** Health economic analysis plan **eAppendix 2.** Resource use questions in the 6-mo follow-up questionnaire booklet **eFigure.** Mean costs of study drugs per participant by day in study **eTable 1.** Sensitivity analysis of costs of study drugs **eTable 2.** Incremental costs and QALYs gained: results of subgroup analyses

Supplement 2.
A2B trial investigators

Supplement 3.
Data Sharing Statement
