# Supplementary material for: Cost-Effectiveness of α2 Agonists for Intravenous Sedation in Patients With Critical Illness
Source: JAMA Netw Open. 2025 May 19;8(5):e2517533. doi: 10.1001/jamanetworkopen.2025.17533 (PMC12090026; doi:10.1001/jamanetworkopen.2025.17533)
Supplement: Supplement 1. — eAppendix 1. Health economic analysis plan eAppendix 2. Resource use questions in the 6-mo follow-up questionnaire booklet eFigure. Mean costs of study drugs per participant by day in study eTable 1. Sensitivity analysis of costs of study drugs eTable 2. Incremental costs and QALYs gained: results of subgroup analyses [file jamanetwopen-e2517533-s001.pdf]

Supplemental Online Content

Morris S, Lone NI, McKenzie CA, Weir CJ, Walsh TS; for the A2B trial investigators. Cost-effectiveness of α_2 agonists for intravenous sedation in patients with critical illness. *JAMA Netw Open*. 2025;8(5):e2517533. doi:10.1001/jamanetworkopen.2025.17533

eAppendix 1. Health economic analysis plan

eAppendix 2. Resource use questions in the 6-mo follow-up questionnaire booklet

eFigure. Mean costs of study drugs per participant by day in study

eTable 1. Sensitivity analysis of costs of study drugs

eTable 2. Incremental costs and QALYs gained: results of subgroup analyses

This supplemental material has been provided by the authors to give readers additional information about their work.

© 2025 Morris S et al. *JAMA Network Open*.

eAppendix 1. Health Economic Analysis Plan

Alpha 2 agonists for sedation to produce better outcomes from critical illness (A2B Trial)

Health Economic Analysis Plan (HEAP)

Prof. Stephen Morris

24 March 2024

Version 2.0

© 2025 Morris S et al. *JAMA Network Open*.

1. Abbreviations

A&E	Accident and emergency
CEAC	Cost-effectiveness acceptability curves
EQ-5D-5L	EuroQoL 5 dimensions 5 level
EVPI	Expected value of perfect information
EVPPI	Expected value of partial perfect information
GP	General practitioner
HEAP	Health economic analysis plan
ICER	Incremental cost-effectiveness ratio
ICU	Intensive Care Unit
MI	Multiple imputation
MICE	Multiple imputation by chained equations
MV	Mechanical ventilation
NHS	National Health Service
NMB	Net monetary benefit
PSS	Personal Social Services
QALY	Quality-adjusted life year
SOFA	Sequential Organ Failure Assessment
VoI	Value of information

2. Study summary

For further details see Walsh *et al.*[1]

Background: Almost all patients receiving mechanical ventilation (MV) in intensive care units (ICUs) require analgesia and sedation. The most widely used sedative drug is propofol, but there is uncertainty whether alpha 2-agonists are superior.

Aim: To determine whether clonidine and/or dexmedetomidine are clinically and cost-effective in MV ICU patients compared with usual care (propofol).

Study design: Open-label three arm randomised trial comparing propofol versus clonidine versus dexmedetomidine as primary sedative, plus analgesia according to local practice.

Participants: 1437 patients (479 per group) from 40–50 UK ICUs. Participants are adult ICU patients within 48 hours of starting MV, expected to require at least 24 hours further MV. Exclusions include patients with primary brain injury; postcardiac arrest; other neurological conditions; or bradycardia.

Primary outcome: Time to successful extubation.

Secondary outcomes: ICU outcomes include delirium and coma incidence/duration, sedation quality, predefined adverse events, mortality and ICU length of stay. Post-ICU outcomes include mortality, anxiety and depression, post-traumatic stress, cognitive function and health-related quality of life at 6-month follow-up.

Analysis: Stage 1 tests whether each alpha 2-agonist is superior to propofol. If either/both interventions are superior, stages 2 and 3 testing explores which alpha 2-agonist is more effective.

[1] Walsh TS, Aitken LM, McKenzie CA, et al. Alpha 2 agonists for sedation to produce better outcomes from critical illness (A2B Trial): protocol for a multicentre phase 3 pragmatic clinical and cost-effectiveness Randomised trial in the UK. *BMJ Open* 2023;13:e078645. doi:10.1136/ bmjopen-2023-078645

© 2025 Morris S et al. *JAMA Network Open*.

3. Purpose of this HEAP

This HEAP describes the health economic analyses that form part of the A2B study, referred to in section 10 of the A2B protocol Version 7.0, dated 25th April 2023. These analyses are pre-specified in order that they are not affected by the collected trial data after unmasking.

4. Aim of this health economic analysis

The aim is to evaluate the cost-effectiveness of clonidine and dexmedetomidine compared with propofol in MV ICU patients from UK NHS and personal social services (PSS) perspective, using individual level data collected in A2B.

5. Overview of health economic analysis

Our analysis will conform to accepted economic evaluation methods in the UK. [2] We will compare dexmedetomidine and clonidine and propofol in full incremental analyses. We will estimate costs and cost-effectiveness over two time horizons: the 'within-trial' period (6 months/short-run model); and, over 5 years following discharge (5 year/long-run model). Costs will be assessed from the perspective of the NHS and PSS. For both the within-trial and long-run analyses we will undertake cost-utility analyses, estimating incremental cost per quality-adjusted life year (QALY) gained. All costs will be reported in 2022/2023 UK£.

6. Identification and measurement of resources

For the within-trial analysis we will calculate detailed cost information on index hospitalisation for every patient from ICU admission to hospital discharge and during 6 months post-randomisation follow-up. Patient-level hospital resource use data for the index hospitalisation will be collected on:

- Use of sedative/analgesic drugs;
- Use of rescue medications for agitation;
- Number of days of MV;
- Length of ICU stay; and,
- Length of hospital ward stay.

Patient-level resource use data will be collected post-discharge up to 6 months post-randomisation using questionnaires at 3 and 6 months on:

- GP contacts at the GP surgery, at home and by telephone;
- District nurse contacts;
- Practice nurse contacts;
- NHS physiotherapist contacts;
- Occupational therapist contacts;
- Speech therapist contacts;
- Dietitian contacts;
- Homecare worker contacts;
- Social worker contacts;
- Psychological therapist contacts;

---

[2] NICE. *NICE health technology evaluations: the manual*. October 2023. https://www.nice.org.uk/process/pmg36/chapter/introduction-to-health-technology-evaluation

© 2025 Morris S et al. *JAMA Network Open*.

- Aids and adaptation worker contacts;
- Substance misuse nurse contacts;
- Macmillan nurse contacts;
- A&E department visits;
- Outpatient visits;
- Hospital re-admissions;
- Rehabilitation hospital stays;
- Day hospital stays; and,
- Nursing home and residential home stays.

In addition the following costs borne by participants and family and friends will be reported but not included in the cost-utility analysis:

- Receipt of unpaid help from family and friend;
- Employment status and return to work; and,
- Out-of-pocket expenditure on health care.

7. Unit costs

All resource use relevant to the NHS and PSS perspective will be valued using UK unit costs (in 2022/2023 £). Unit costs will be identified from published sources (the *British National Formulary*[3], the drugs and pharmaceutical electronic market information tool (eMIT)[4], *Unit Costs of Health and Social Care*[5], *Prescription Cost Analysis*[6], and the *National Schedule of NHS Costs*[7]). Days in ICU will be costed according to the number of organs supported, which will be identified each day from the trial data collected on daily SOFA scores. A table of unit costs, together with their sources, will be produced and inflated to 2022/2023 prices using NHS Pay and Prices Indices[4] for each base year.

8. Total costs

The cost of all reported resource use (relevant to an NHS and PSS perspective) will be calculated for each participant. These figures will then be summed for each participant, giving a total cost over the 6-month within-trial time horizon period.

9. Reporting of resource use and cost data

Mean (std.dev.) resource use per participant will be estimated for each item of resource use listed above for each randomised group. Mean differences (95% CI) in mean resource use between dexmedetomidine and clonidine versus propofol, and between dexmedetomidine and clonidine, will be presented (unadjusted for confounders). Mean (std.dev.) total costs per participant will be estimated for each randomised group. Mean difference (95% CI) in mean total costs between dexmedetomidine and clonidine versus propofol, and between dexmedetomidine and clonidine, will be presented (unadjusted for confounders). These data will not be adjusted for missing data.

---

[3] https://bnf.nice.org.uk/
[4] https://www.gov.uk/government/publications/drugs-and-pharmaceutical-electronic-market-information-emit
[5] https://www.pssru.ac.uk/project-pages/unit-costs/
[6] https://www.nhsbsa.nhs.uk/prescription-data/dispensing-data/prescription-cost-analysis-pca-data
[7] https://www.england.nhs.uk/national-cost-collection/

© 2025 Morris S et al. *JAMA Network Open*.

10. Identification of outcomes

The primary economic analysis outcome measure will be quality-adjusted life-years (QALYs) estimated using utility scores obtained using the EQ-5D-5L instrument[8] collected at 30 days, 3 and 6 months. As patients recruited to the trial will be critically ill, completion of the EQ-5D-5L at baseline will not be possible. In the base case we will calculate QALYs assuming a baseline utility score of zero.[9] Participants were also asked to retrospectively record their baseline EQ-5D-5L at the 30 day follow-up point, and baseline EQ-5D-5L scores for participants were also recorded by proxy respondents. In sensitivity analysis we will recalculate QALYs using these two alternative measures. We will assign all the EQ-5D-5L data that are collected to either the baseline, 30 days, 3 or 6 months measurement points, irrespective of the precise time when these were actually measured.

11. Measurement of QALYs

Responses to the EQ-5D-5L will be converted to utility scores using UK preference weights in line with recommendations at the time of analysis (currently the Hernandez-Alava algorithm[10]). We will evaluate QALYs associated with each randomised group. Patients who die will be assigned a utility value of zero at the date of death and all subsequent time periods. Patient-specific utility profiles will be constructed assuming a straight line relation between each of the patients' EQ-5D-5L scores at each follow-up point. The QALYs experienced by each patient from baseline to 6 months will be calculated as the area underneath this profile, computed as the ((utility score at baseline + utility score at 30 days)/24) + ((utility score at 30 days + utility score at 3 months)/12) + ((utility score at 3 months + utility score at 6 months)/8).

12. Reporting of utility scores and QALYs

Mean (std.dev.) utility scores per participant at each follow-up point will be estimated for each randomised group. Mean difference (95% CI) in mean utility scores between dexmedetomidine and clonidine versus propofol, and between dexmedetomidine and clonidine, will be presented (unadjusted for confounders). Mean (std.dev.) QALYs per participant will be estimated for each randomised group. Mean difference (95% CI) in mean QALYs between dexmedetomidine and clonidine versus propofol, and between dexmedetomidine and clonidine, will be presented (unadjusted for confounders). These data will not be adjusted for missing data.

13. Time horizon and discounting

Costs and outcomes will be valued over a 6-month time horizon for the within-trial analysis. Costs and outcomes will not be discounted given that the time horizon for this analysis is 6 months.

14. Missing data

We will summarise the amount of missing data for resource use, costs and outcomes, and use regression analysis to identify participant characteristics associated with missing data. Multiple imputation (MI) will

---

[8] https://euroqol.org/eq-5d-instruments/eq-5d-5l-about/

[9] Improve Trial Investigators. Endovascular strategy or open repair for ruptured abdominal aortic aneurysm: one-year outcomes from the IMPROVE randomized trial. *Eur Heart J* 2015;36:2061-9. https://doi.org/10.1093/eurheartj/ehv125

[10] Hernández-Alava M, Pudney S. eq5dmap: A command for mapping between EQ-5D-3L and EQ-5D-5L. *The Stata Journal* 2018;18:395-415. https://www.stata-journal.com/article.html?article=st0528

© 2025 Morris S et al. *JAMA Network Open*.

be performed assuming missingness at random to replace each missing observation with a set of imputed values following the method recommended by Faria et al.[11] for the imputation of economic data. We will use multiple imputation by chained equations (MICE), and Rubin's rules[12] will be implemented for the subsequent analysis of multiple datasets.

15. Measuring cost-effectiveness

Costs and outcomes will be calculated per participant and then analysed using generalised linear models controlling for covariates to estimate incremental costs and QALYs gained for participants receiving dexmedetomidine and clonidine versus propofol. Covariates considered will be the same as for the main statistical analysis, accounting for study site; we will also adjust for baseline utility score in the analysis of QALYs.[13] Incremental cost-effectiveness ratios (ICERs), and also incremental net monetary benefits (NMBs) computed at willingness-to-pay thresholds of £13,000, £20,000 and £30,000 per QALY, will be calculated for dexmedetomidine and clonidine versus propofol, and for dexmedetomidine versus clonidine. Non-parametric bootstrapping with 5,000 replications to accommodate sampling uncertainty will be undertaken. The cost-effectiveness estimates (ICERs, incremental NMBs) will be derived using the data from these probabilistic analyses. The probability of each option (dexmedetomidine, clonidine, propofol) being cost-effective based on it having the highest NMB at different cost-effectiveness thresholds will be calculated[14] and represented visually as cost-effectiveness acceptability curves (CEACs).[15]

16. Reporting cost-effectiveness

Analyses will be performed on an intention-to-treat basis and following the reference case outlined in NICE guidelines.[16] The probability that each option is cost-effective at thresholds of £13,000, £20,000 and £30,000 per QALY will be reported for (1) the base case (missing values imputed using MI, with adjustment for potential confounders), (2) with missing values imputed using MI but with no adjustment for potential confounders, (3) with no MI but adjustment for potential confounders, and (4) with no MI or adjustment for potential confounders.

We will report cost-effectiveness findings for subgroups of participants, using identical sub-groups to those described in the statistical analysis plan, as follows:

- Patients with and without sepsis at enrolment.
- Patients with lower or higher delirium risk, as defined by values of the PRE-DELIRIC delirium risk prediction score[17] above (or including) and below the median.
- Patients with and without organ dysfunction at randomisation, as defined by SOFA score values above or equal to the median and below the median.

---

[11] Faria R, et al. A guide to handling missing data in cost-effectiveness analysis conducted within randomised controlled trials. *Pharmacoeconomics*. 2014;32(12):1157–70.
[12] Little RJ, Rubin DB. *Statistical analysis with missing data*, vol. 333. Hoboken: Wiley; 2014.
[13] Manca A, Hawkins N, Sculpher MJ. Estimating mean QALYs in trial-based cost-effectiveness analysis: the importance of controlling for baseline utility. *Health Econ*. 2005;14(5):487–96.
[14] Fenwick E, Claxton K, Sculpher M. Representing uncertainty: the role of cost-effectiveness acceptability curves. *Health Econ*. 2001;10(8):779–87.
[15] van Hout BA, et al. Costs, effects and C/E-ratios alongside a clinical trial. *Health Econ*. 1994;3(5):309–19.
[16] https://www.nice.org.uk/process/pmg36/resources/nice-health-technology-evaluations-the-manual-pdf-72286779244741
[17] van den Boogaard M, Pickkers P, Slooter AJ, et al. Development and validation of PRE-DELIRIC (PREdiction of DELIRium in ICu patients) delirium prediction model for intensive care patients: observational multicentre study. *BMJ* (Clinical research ed) 2012;344:e420. doi: 10.1136/bmj.e420

© 2025 Morris S et al. *JAMA Network Open*.

- Age (<64 versus ≥64)

17. Sensitivity analysis

As noted, CEACs will be generated via non-parametric bootstrapping with 5,000 replications to accommodate sampling uncertainty and varying levels of the cost-effectiveness willingness to pay threshold from £0 to £50,000 for an additional QALY.

Univariate deterministic sensitivity analyses (varying one variable at a time) will examine the impact on the cost-effectiveness results of varying the drug costs of dexmedetomidine and clonidine, and volume of resource use and unit cost for each cost component, and utility scores. As noted, we will recalculate QALYs assuming a baseline utility score of zero and explore the implications of this on cost-effectiveness. We will also calculate the unit cost of dexmedetomidine and clonidine required, for both drugs to be cost-effective versus usual care (i.e., for the ICER to equal the cost-effectiveness threshold).

18. Statistical packages

All analyses will be performed using Stata software. [18]

19. Long-run analysis

In the long-run analysis cost-effectiveness will be calculated in terms of the incremental cost per QALY gained. Survival and hospital costs up to 5 years will be predicted for individual trial participants alive at 6 months follow-up, using published statistical models that account for participant characteristics. [19] Utility scores at 6 months from the within-trial analysis will be applied to survival over 5 years to calculate long-run QALYs. As a sensitivity analysis we will use utility scores from ICU survivors from published sources to calculate long-run QALYs. [20] The ratio of hospital costs to non-hospital NHS and PSS costs from the within-trial analysis over 6-months post-discharge will be applied to predicted hospital costs over 5 years to model total NHS and PSS costs over 5 years.

Long-run ICERs and incremental NMBs will be calculated at willingness-to-pay thresholds of £13,000, £20,000 and £30,000 per QALY for dexmedetomidine and clonidine versus propofol, and for dexmedetomidine versus clonidine. We will undertake deterministic (one-, two- and multi-way) and probabilistic sensitivity analysis, assuming appropriate distributions for all parameters. The probability of each option (dexmedetomidine, clonidine, propofol) being cost-effective based on it having the highest NMB at different cost-effectiveness thresholds will be calculated and represented visually as CEACs.

We will also undertake value-of-information (VoI) analyses, based on the notion that investing in further research on probabilities of events, utility scores and costs will reduce decision uncertainty about the cost-effectiveness of dexmedetomidine and clonidine versus propofol. These will include both the expected value of perfect information (EVPI) and the expected value of partial perfect information (EVPPI). The latter focusing on individual model parameters or groups of parameters [21].

---

[18] StataCorp. 2017. Stata Statistical Software: Release 15. College Station, TX: StataCorp LLC.
[19] Lone NI, Gillies MA, Haddow C, Dobbie R, Rowan KM, Wild SH, Murray GD, Walsh TS. Five-Year Mortality and Hospital Costs Associated with Surviving Intensive Care. *Am J Respir Crit Care Med.* 2016 Jul 15;194(2):198-208. doi: 10.1164/rccm.201511-2234OC.
[20] Cuthbertson, B.H., Roughton, S., Jenkinson, D. et al. Quality of life in the five years after intensive care: a cohort study. *Crit Care* 14, R6 (2010). https://doi.org/10.1186/cc8848
[21] Briggs, A., M. Sculpher, and K. Claxton, Decision modelling for health economics evaluation. Oxford University Press, 2006.

© 2025 Morris S et al. *JAMA Network Open*.

20. Reporting

A Consolidated Health Economic Evaluation Reporting Standards 2022 (CHEERS 2022) checklist will be reported,[22] which makes recommendations as to what ought to be reported in an economic evaluation across 28 items in 7 categories (title, abstract, introduction, methods, results, discussion, other relevant information). This requires a brief summary of how each aspect is reported and details of where it is reported in the text.

[22] Husereau D, Drummond M, Augustovski F, de Bekker-Grob E, Briggs A H, Carswell C et al. Consolidated Health Economic Evaluation Reporting Standards 2022 (CHEERS 2022) statement: updated reporting guidance for health economic evaluations *BMJ* 2022; 376 :e067975 doi:10.1136/bmj-2021-067975

eAppendix 2. Resource use questions in the 6 month follow-up questionnaire booklet

© 2025 Morris S et al. *JAMA Network Open*.

An ICU Sedation Study

6 month Follow-up Questionnaire Booklet

Study ID : ______________

Date Completed: ___________________

- Thank you for agreeing to take part in this study
- This booklet contains four questionnaires
- A researcher will try to call you to complete a fifth questionnaire over the phone
- Instructions for completion are shown at the start of each questionnaire
- Please answer all the questions
- Return this booklet by post in the pre-paid envelope provided
- If you have any difficulties or questions please refer to the accompanying letter for details of who to contact for advice

Thank you for your support with this study

FUNDED BY
NIHR | National Institute for Health Research

This study is funded by the National Institute for Health Research (NIHR) Health Technology Assessment (HTA) Programme (16/93/01). The views expressed are those of the author(s) and not necessarily those of the NIHR or the Department of Health and Social Care.

© 2025 Morris S et al. *JAMA Network Open*.

An ICU Sedation Study

6 MONTH HEALTH SERVICE UTILISATION QUESTIONNAIRE

HOW TO FILL IN THIS QUESTIONNAIRE

Please try to complete the whole questionnaire. You may not be able to exactly remember the answer to some questions but please give your best estimate.

The questions relate to the time since the 3 month follow-up. If you have not left hospital since you were in intensive care please answer questions 1 and 2 only.

Please print carefully within the boxes like this

2	7

or like this

X

© 2025 Morris S et al. *JAMA Network Open*.

THIS SET OF QUESTIONS IS ABOUT YOUR EMPLOYMENT

If you were in paid employment before your hospital admission, please answer the questions below. If not please go to question 3.

1. Have you returned to work?

Yes ☐

No ☐

2. Are you working

full time ☐

part time ☐

IF YOU HAVE NOT LEFT HOSPITAL SINCE YOU WERE IN INTENSIVE CARE THEN YOU HAVE COMPLETED THIS QUESTIONNAIRE AND DO NOT NEED TO ANSWER ANY FURTHER QUESTIONS

THIS SET OF QUESTIONS IS ABOUT ANY UNPAID HELP YOU HAVE RECEIVED FROM A FAMILY MEMBER (E.G. SPOUSE OR PARTNER) OR FRIEND SINCE THE 3 MONTH FOLLOW-UP

3. Have you received any unpaid help from a family member (e.g. spouse or partner) or friend?

Yes ☐

No ☐

(IF YOU ANSWERED NO THEN PLEASE GO TO QUESTION 9)

If Yes, please give details about your main helper below:

4. Was your main helper in paid employment before the illness that caused your intensive care admission?

Yes ☐

No ☐

(IF YOU ANSWERED NO THEN PLEASE GO TO QUESTION 9)

© 2025 Morris S et al. *JAMA Network Open*.

If Yes, how many paid hours a week do they currently work?

0-5 ☐ **6-15** ☐ **16-25** ☐ **26-35** ☐ **over 35** ☐

5. Have they been given time off paid work to help you during our recovery? Yes ☐ No ☐

6. Have they reduced their paid working hours to help you during your recovery? Yes ☐ No ☐

7. Have they had to change their job to help you during your recovery? Yes ☐ No ☐

8. Have they given up paid employment completely to help you during your recovery? Yes ☐ No ☐

THIS SET OF QUESTIONS IS ABOUT ANY APPOINTMENTS YOU HAVE HAD WITH A GP SINCE THE 3 MONTH FOLLOW-UP

9. Have you seen a GP for any reason since the 3 month follow-up? Yes ☐ No ☐

If Yes, please give details below:

10. How many times have you visited a GP? ☐☐

11. How many times have you had a GP visit you at home? ☐☐

12. How many times have you had a telephone conversation with a GP? ☐☐

© 2025 Morris S et al. *JAMA Network Open*.

THIS SET OF QUESTIONS IS ABOUT ANY OTHER HEALTH CARE WORKERS YOU HAVE SEEN SINCE THE 3 MONTH FOLLOW-UP

13. Since the 3 month follow-up have you been seen by

**A district nurse?**	**Yes** ☐ **No** ☐	**If Yes, how many times?**	☐☐
**A practice nurse?**	**Yes** ☐ **No** ☐	**If Yes, how many times?**	☐☐
**An NHS physiotherapist?**	**Yes** ☐ **No** ☐	**If Yes, how many times?**	☐☐
**An occupational therapist?**	**Yes** ☐ **No** ☐	**If Yes, how many times?**	☐☐
**A speech therapist?**	**Yes** ☐ **No** ☐	**If Yes, how many times?**	☐☐
**A dietitian?**	**Yes** ☐ **No** ☐	**If Yes, how many times?**	☐☐
**A homecare worker?** e.g. meals on wheels	**Yes** ☐ **No** ☐	**If Yes, how many times?**	☐☐
**A social worker?**	**Yes** ☐ **No** ☐	**If Yes, how many times?**	☐☐
**A psychological therapist?** e.g. psychologist, psychiatrist, psychology counsellor	**Yes** ☐ **No** ☐	**If Yes, how many times?**	☐☐
**A counsellor?**	**Yes** ☐ **No** ☐	**If Yes, how many times?**	☐☐
**A day hospital?**	**Yes** ☐ **No** ☐	**If Yes, how many times?**	☐☐
**An aids and adaptations worker?** (a person who has provided aids or adaptations)	**Yes** ☐ **No** ☐	**If Yes, how many times?**	☐☐
**A substance misuse nurse?**	**Yes** ☐ **No** ☐	**If Yes, how many times?**	☐☐
**A MacMillan nurse?**	**Yes** ☐ **No** ☐	**If Yes, how many times?**	☐☐
**other *(please specify)*** ______	**Yes** ☐ **No** ☐	**If Yes, how many times?**	☐☐

© 2025 Morris S et al. *JAMA Network Open*.

THIS QUESTION IS ABOUT ANY ACCIDENT AND EMERGENCY VISITS YOU HAVE HAD SINCE THE 3 MONTH FOLLOW-UP

14. Have you been to accident and emergency since the 3 month follow-up?

Yes ☐

No ☐

If Yes, how many times did you go? ☐☐

THIS QUESTION IS ABOUT ANY HOSPITAL APPOINTMENTS YOU HAVE HAD SINCE THE 3 MONTH FOLLOW-UP

15. Have you been to any hospital clinics since the 3 month follow-up?

Yes ☐

No ☐

If Yes, how many appointments did you go to? ☐☐

THIS QUESTION IS ABOUT ANY HOSPITAL READMISSIONS YOU HAVE HAD SINCE THE 3 MONTH FOLLOW-UP

16. Have you been readmitted to hospital since the 3 month follow-up?

Yes ☐

No ☐

If Yes, please provide details of all readmissions

**Reason for re-admission**	**Number of days in hospital**
1.__________________________________	☐☐
2.__________________________________	☐☐
3.__________________________________	☐☐

© 2025 Morris S et al. *JAMA Network Open*.

THIS QUESTION IS ABOUT ANY STAYS YOU HAVE HAD IN A REHABILITATION HOSPITAL, SINCE THE 3 MONTH FOLLOW-UP

17. Have you spent time in a rehabilitation hospital since the 3 month follow-up?

Yes ☐

No ☐

If Yes, please write down hospital names and days spent in each

Hospital name	Days spent in this hospital
____________________	____________
____________________	____________
____________________	____________
____________________	____________

THIS QUESTION IS ABOUT ANY STAYS YOU HAVE HAD IN A NURSING HOME, RESIDENTIAL CARE (OR SIMILAR) SINCE THE 3 MONTH FOLLOW-UP

18. Have you spent time in a nursing home, residential care (or similar) since the 3 month follow-up?

Yes ☐

No ☐

If Yes, please write down hospital/home names and days spent in each

Hospital/home name	Days spent in this hospital/home
____________________	____________
____________________	____________
____________________	____________
____________________	____________

© 2025 Morris S et al. *JAMA Network Open*.

THIS QUESTION ASKS ABOUT ANY MONEY YOU HAVE HAD TO SPEND OUT OF YOUR OWN POCKET ON HEALTH CARE SINCE THE 3 MONTH FOLLOW-UP

19. Have you had to spend any of your own money on health care (for example on medications, or visits to private practitioners e.g. physiotherapists or complimentary therapists) since the 3 month follow-up?

Yes ☐

No ☐

If Yes, please provide us with details and an approximate figure (to the nearest £)

____ Cost in £ _____

© 2025 Morris S et al. *JAMA Network Open*.

eFigure. Mean costs of study drugs per participant by day in study

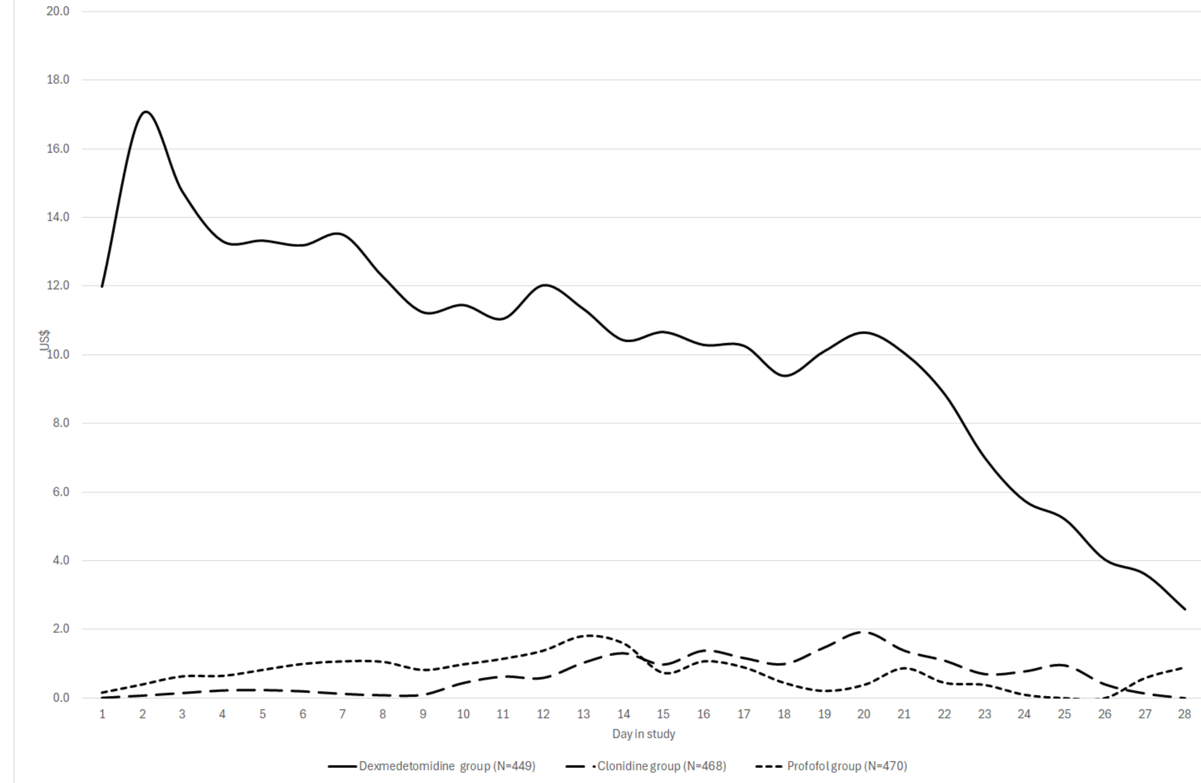

(a) Daily dexmedetomidine costs

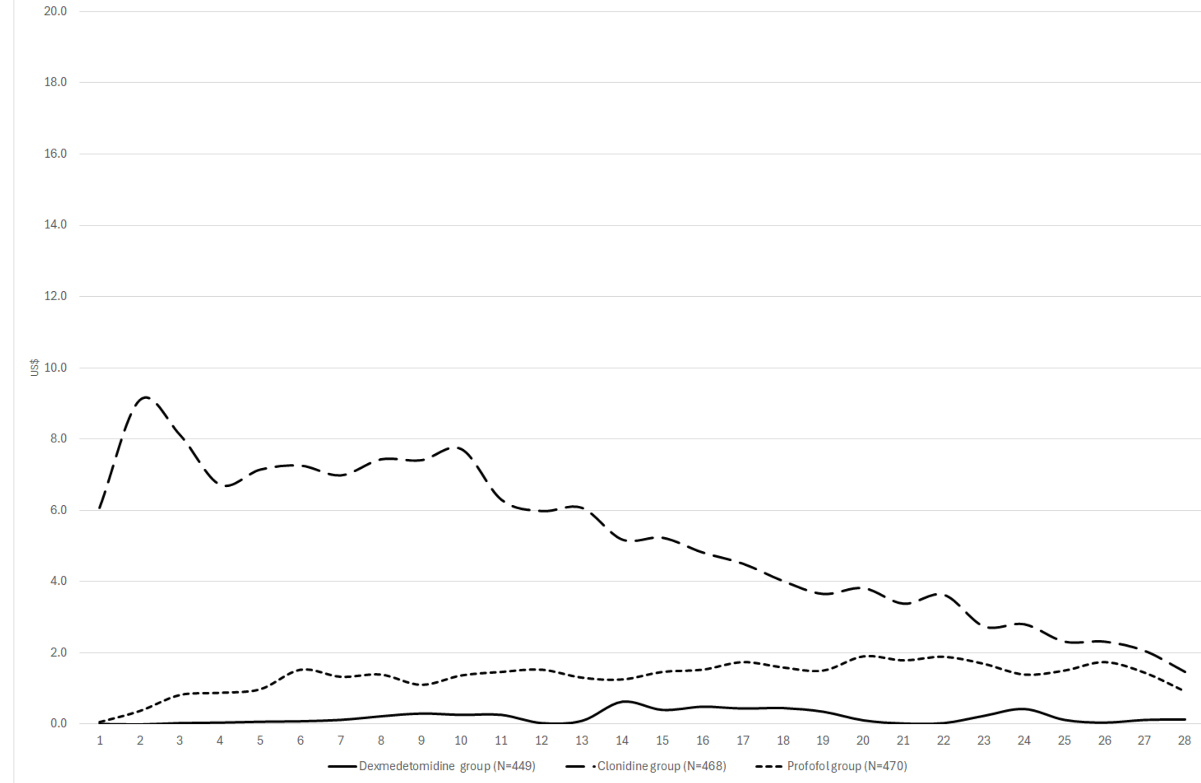

(b) Daily clonidine costs

© 2025 Morris S et al. *JAMA Network Open*.

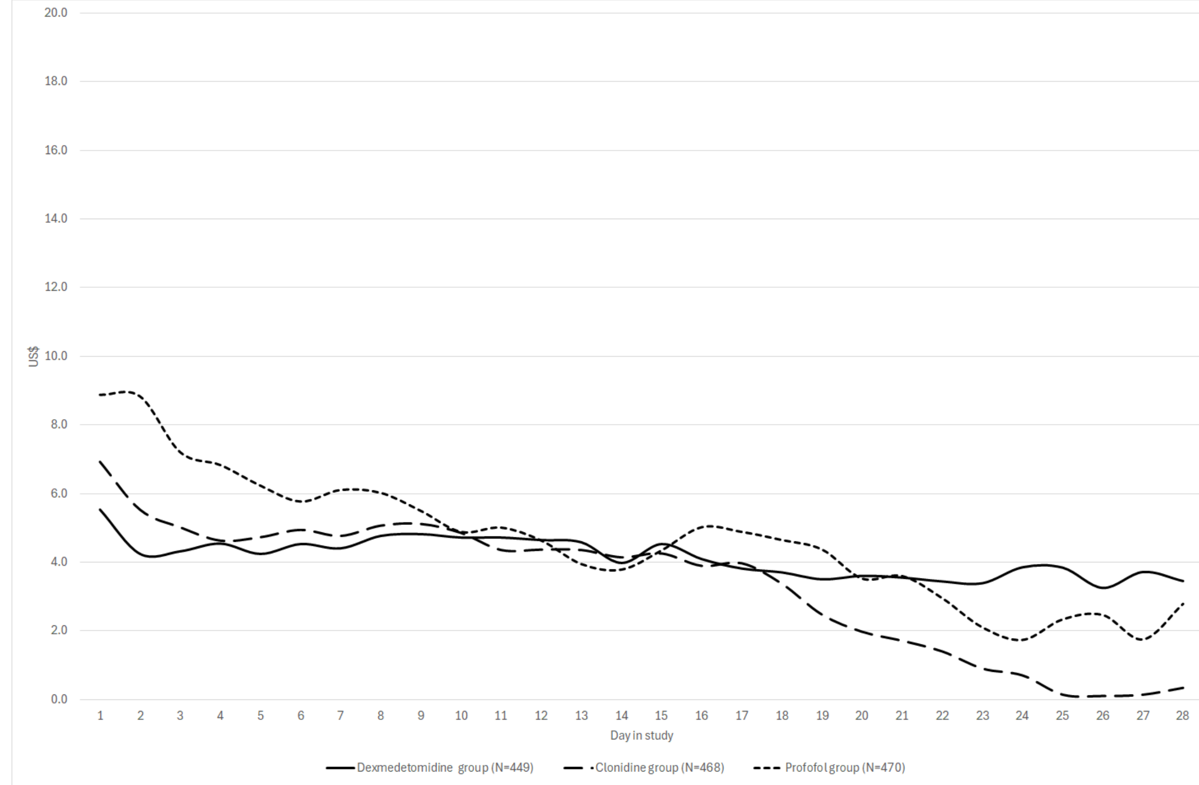

(c) Daily propofol costs

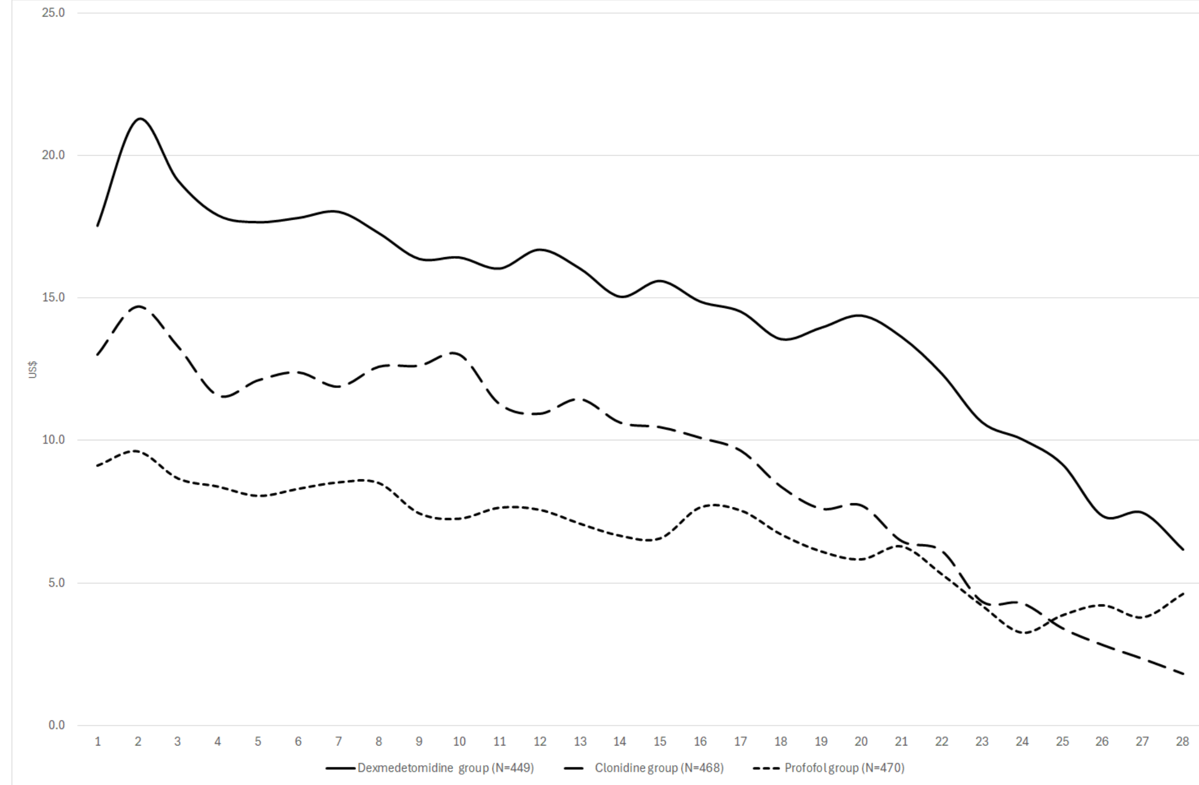

(d) Daily combined costs of study drugs

Costs are based on quantities of study drugs recorded up to achievement of primary outcome (successful extubation), truncated at day 28 after admission to the ICU. Costs are in 2023/24 US$ (UK£1=US$1.25).[13]

© 2025 Morris S et al. *JAMA Network Open*.

eTable 1. Sensitivity analysis of costs of study drugs

	Dexmedetomidine (Obs=449)		Clonidine (Obs=468)		Propofol (Obs=470)		
	Mean	(SD)	Mean	(SD)	Mean	(SD)	Unit costs per day, US$
**Number of days receiving study drugs and costs of study drugs**							
**Days**							
Dexmedetomidine	6.5	(5.4)	0.2	(1.1)	0.4	(1.7)	
Clonidine	0.2	(0.9)	6.3	(5.3)	1.1	(3.0)	
Propofol	5.8	(5.4)	5.9	(5.1)	6.7	(5.3)	
**Costs, US$**							
Dexmedetomidine	178	(149)	5	(31)	11	(46)	28
Clonidine	1	(9)	64	(53)	11	(30)	10
Propofol	108	(100)	113	(95)	125	(100)	19
Combined	288	(235)	178	(148)	146	(133)	

SD = standard deviation. Costs are in 2023/24 US$ (UK£1=US$1.25).[13]

© 2025 Morris S et al. *JAMA Network Open*.

eTable 2. Incremental costs and QALYs gained: results of sub-group analyses

	Incremental cost, US$	QALYs gained
	**Mean (95% CI)**	**Mean (95% CI)**
**Sub-group**		
Age<64 years		
Dexmedetomidine vs. Propofol	3494 (-5248 to 12237)	0.0025 (-0.0258 to 0.0307)
Clonidine vs. Propofol	-1180 (-9081 to 6277)	0.0020 (-0.0247 to 0.0287)
Age≥64 years		
Dexmedetomidine vs. Propofol	-1823 (-10808 to 7162)	-0.00183 (-0.0351 to 0.0315)
Clonidine vs. Propofol	-1421 (-9972 to 7130)	-0.0054 (-0.0358 to 0.0249)
Without sepsis at enrolment		
Dexmedetomidine vs. Propofol	-1204 (-11309 to 8900)	0.0029 (-0.0342 to 0.0399)
Clonidine vs. Propofol	-4515 (-14304 to 5274)	-0.0167 (-0.0528 to 0.0194)
With sepsis at enrolment		
Dexmedetomidine vs. Propofol	1790 (-6212 to 9791)	0.0006 (-0.0253 to 0.0266)
Clonidine vs. Propofol	258 (-6901 to 7418)	0.0069 (-0.0175 to 0.0315)
Below the median baseline SOFA score		
Dexmedetomidine vs. Propofol	3919 (-6840 to 14678)	-0.0069 -0.0413 to 0.0275)
Clonidine vs. Propofol	-1327 (-10161 to 7508)	-0.0008 -0.0355 to 0.0337)
Above the median baseline SOFA score		
Dexmedetomidine vs. Propofol	607 (-7647 to 8861)	0.0039 (-0.0238 to 0.0317)
Clonidine vs. Propofol	-2600 (-10244 to 5044)	-0.0054 (-0.0319 to 0.0211)
Below the median baseline PRE-DELIRIC delirium risk prediction score		
Dexmedetomidine vs. Propofol	916 (-8331 to 10162)	0.0015 (-0.0277 to 0.0308)
Clonidine vs. Propofol	-785 (-9249 to 7678)	-0.0083 (-0.0377 to 0.0210)
Above the median baseline PRE-DELIRIC delirium risk prediction score		
Dexmedetomidine vs. Propofol	1059 (-7734 to 9853)	0.0069 (-0.0231 to 0.0370)
Clonidine vs. Propofol	-2719 (-10850 to 5411)	0.0106 (-0.0179 to 0.0393)

QALY = quality-adjusted life year. CI = confidence interval. SOFA = Sequential Organ Failure Assessment. PRE-DELIRIC = PREdiction of DELIRium in ICu patients. Costs are in 2023/24 US$ (UK£1=US$1.25).[13] All analysis are undertaken using base case assumptions. Data include values imputed using multiple imputation (see text) with adjustment for study site.

© 2025 Morris S et al. *JAMA Network Open*.
