# Supplementary material for: Cost-Effectiveness of α2 Agonists for Intravenous Sedation in Patients With Critical Illness
Source: JAMA Netw Open. 2025 May 19;8(5):e2517533. doi: 10.1001/jamanetworkopen.2025.17533 (PMC12090026; doi:10.1001/jamanetworkopen.2025.17533)
Supplement: Supplement 2. — A2B trial investigators [file jamanetwopen-e2517533-s002.pdf]

*Group Name(s): A2B trial investigators							
***First Name and Middle Initial(s)**	***Last Name**	***Suffix (eg, Jr, III)**	Academic Degrees	Institution	Location (city, state/province, country)	Role or Contribution, eg, chair, principal investigator	Group (if more than 1 Group listed in the byline) and/or Subgroup (eg, Steering Committee)
Natalie	Pattison						
Barry	Williams						
Louise	Rose						
Paul	Mouncey						
John	Prowle						
David	Wellsted						
Tim	Walsh						
Stephen	Brett						
Heather	Charles						
Christoper	Weir						
John	Norrie						
Julian	Bion						
Graeme	McLennan						
Matt	Stevenson						
Alistair	Nichol						
Timothy	Walsh						
Maria	Amamio						
Lucy	Barclay						
Sophie	Birch						
Kate	Briton						
Sarah	Clark						
Jessica	Crossan						
Katherine	Doverman						
David	Hope						
Lucy	Macdonald						
Corrienne	McCulloch						
Nicola	Rae						
Scott	Simpson						
Jo	Singleton						

***First Name and Middle Initial(s)**	***Last Name**	***Suffix (eg, Jr, III)**	Academic Degrees	Institution	Location (city, state/province, country)	Role or Contribution, eg, chair, principal investigator	Group (if more than 1 Group listed in the byline) and/or Subgroup (eg, Steering Committee)
Maggie	Wishart						
Ruth	Thompson						
Neill	Aitken						
Rachel	Fairlie						
Nabeel	Salim						
Sam	Talbot						
Sarah	Ackroyd						
Valeria	Alicino						
Euan	Allan						
Thomas	Anderson						
Rosemary	Andrew						
Andrew	Baigey						
Kenneth	Baillie						
Calum	Barnetson						
Ruth	Begbie						
Richie	Biggers						
Michael	Blaney						
Richard	Broom						
David	Birrell						
Will	Calkin						
Keegan	Chuavilong						
Rebecca	Cowden						
Thomas	Cox						
Coral	Darjee						
Simon	Davies						
Annemarie	Docherty						
Luke	Dornan						
Mark	Dunn						
Stuart	Edwardson						
Ross	Gillespie						
Jane	Greenwood						

***First Name and Middle Initial(s)**	***Last Name**	***Suffix (eg, Jr, III)**	Academic Degrees	Institution	Location (city, state/province, country)	Role or Contribution, eg, chair, principal investigator	Group (if more than 1 Group listed in the byline) and/or Subgroup (eg, Steering Committee)
David	Griffith						
Alasdair	Hay						
Amy	Hu						
Ali	Hunter						
Karen	Jones						
Helen	Jordan						
Ancy	Joseph						
Kallirroi	Kefala						
Stephanie	Kelly						
Laura	Kemp						
Bara	Kubanova						
Victoria	Leng						
John	Livesey						
Nazir	Lone						
James	Lyon						
Olivia	Mansfield						
Dean	McAvoy						
Aaron	McClatchey						
Jonathan	Miller						
Ananda	Mirchandani						
Peter	Moffitt						
Steven	Morrison						
Alexandra	Muir						
Kieran	Nunn						
John	Ochiltree						
Emily	Ogden						
Matthew	Parks						
Marc	Pass						
Rachael	Penrose						
Harry	Putnam						
Thomas	Quinn						

***First Name and Middle Initial(s)**	***Last Name**	***Suffix (eg, Jr, III)**	Academic Degrees	Institution	Location (city, state/province, country)	Role or Contribution, eg, chair, principal investigator	Group (if more than 1 Group listed in the byline) and/or Subgroup (eg, Steering Committee)
Jonathan	Rhodes						
Alexander	Rollings						
Stephen	Ross						
Ralph	Shackleton						
Manu	Shankar						
Sunil	Sharma						
Iain	Slessor						
Zack	Slevin						
Duncan	Stickle						
Louise	Symons						
Fiona	Walker						
Luke	Walls						
Ian	Whiteford						
Sue Yin	Yong						
Neil	Young						
Kevin	Rooney						
Michael	Kinsella						
Brian	Digby						
Michael	Brett						
Paul	McConnell						
Mark	Henderson						
Radha	Sundaram						
Lisa	Gemmell						
Fiona	Christie						
Philip	Henderson						
Fiona	MacGregor						
Steven	Henderson						
Natalie	Rodden						
Kirsty	Fallon						
Lynn	Abel						
Barbara	McLaren						

***First Name and Middle Initial(s)**	***Last Name**	***Suffix (eg, Jr, III)**	Academic Degrees	Institution	Location (city, state/province, country)	Role or Contribution, eg, chair, principal investigator	Group (if more than 1 Group listed in the byline) and/or Subgroup (eg, Steering Committee)
Emma	Hughes						
Deborah	McGlynn						
Nicola	Thomson						
Lauren	Walker						
Susan	Currie						
Natasha	Parker						
Donna	Gillan						
Farooq	Brohi						
Sarah	Purvis						
Michele	Clark						
Pam	Race						
Lynne	Williams						
Ahmed	Shahin						
Eusebius	Nworah						
Jonathan	Gui						
Li-Chin	Cheng						
Katelyn	Stewart						
Rebecca	Cusack						
Mark	Tomlin						
Clare	Bolger						
Rachel	Burnish						
Sue	Jackson						
Alice	Baker						
Jonathan	Biss						
Karen	Salmon						
Michael	Carter						
Catherine	McKenzie						
Razaz	Elsheikh						
Missy (Anne)	Harrison						
Charlotte	Thomas						
James	Ward						

***First Name and Middle Initial(s)**	***Last Name**	***Suffix (eg, Jr, III)**	Academic Degrees	Institution	Location (city, state/province, country)	Role or Contribution, eg, chair, principal investigator	Group (if more than 1 Group listed in the byline) and/or Subgroup (eg, Steering Committee)
Andrew	Cumpstey						
Ahilanandan	Dushianthan						
Ivan	Kemp						
Valerie	Page						
Xiaobei	Zhao						
Nazril	Nordin						
Ahmed	Hegazy						
Elvira	Hoxha						
Owen	Hardaker						
Chimenime	Ede						
Nailia	Kotrikova						
Acharya	Devaraja						
Thomas	Stambach						
Prasun	Mukherjee						
Mark Louie	Guanco						
Matthew P	Wise						
Jade	Cole						
Helen	Hill						
Jenny	Brooks						
Michelle	Davies						
Rhys	Davies						
Emma	Thomas						
Angharad	Williams						
Lauren	Lodhi						
Matt PG	Morgan						
Simon	Ridler						
Christopher	Smith						
Maria	Faulkner						
Alison	Ivison						
Laura	McKay						
Helen	Jeffrey						

***First Name and Middle Initial(s)**	***Last Name**	***Suffix (eg, Jr, III)**	Academic Degrees	Institution	Location (city, state/province, country)	Role or Contribution, eg, chair, principal investigator	Group (if more than 1 Group listed in the byline) and/or Subgroup (eg, Steering Committee)
Jude	Price						
Lucy	Slater						
Angela	Davies						
Edward	Hughes						
Matt	Thomas						
Dominic	Janssen						
Ian	Thomas						
Kate	Crewdson						
Christopher	Newell						
Robert	Hirst						
Stephen	West						
Agnieszka	Skorko						
Emma	Gendall						
Ruth	Worner						
Beverley	Faulkner						
Borislava	Borislavova						
Kati	Hayes						
Andrew	Parsons						
Elizabeth	Goff						
John	Sowersby						
Annie	Wood						
Kieran	Oglesby						
Idrisu	Sanusi						
Charlie	Pope						
Andrew	Baird						
Hayley	Blackmore						
Robert	Healey						
Philip	Hopkins						
Eleanor	Corcoran						
Gillian	Selman						
Clare	Finney						

***First Name and Middle Initial(s)**	***Last Name**	***Suffix (eg, Jr, III)**	Academic Degrees	Institution	Location (city, state/province, country)	Role or Contribution, eg, chair, principal investigator	Group (if more than 1 Group listed in the byline) and/or Subgroup (eg, Steering Committee)
Evita	Pappa						
John	Smith						
Emma	Clarey						
Maeve	Cockrell						
Sian	Saha						
Harriet	Noble						
Kevin	O'Reilly						
Maria	Depante						
Anna	Broderick						
Marianette Anne	Axalan						
Burt	Vergara						
Reena	Mehta						
Henrik	Reschrieter						
Sarah	Patch						
Julie	Camsooksai						
Sarah	Jenkins						
Madga	Pomichowska						
Ken	Power						
Spike	Briggs						
Elizabeth	Woodward						
Christopher	Loew						
James	Bromilow						
James	Keegan						
Matthew	Taylor						
Emma	Langridge						
Dinesh	Kulandhaisamy						
Saah	Savage						
Yasmin	de'Ath						
Charlotte	Humphrey						
Sue	Roffe						
Matthew	Bayliss						

***First Name and Middle Initial(s)**	***Last Name**	***Suffix (eg, Jr, III)**	Academic Degrees	Institution	Location (city, state/province, country)	Role or Contribution, eg, chair, principal investigator	Group (if more than 1 Group listed in the byline) and/or Subgroup (eg, Steering Committee)
Leanne	Bartlett						
Richard	Gordon-Williams						
Kate	Tatham						
Sam	Smith						
Isabel	Noris						
Sharjeel	Tahir						
Emma	Yates						
Shivali	Patel						
Tanith	Westerman						
Sekina	Bakare						
Hugh	Furness						
Emma	Hunt						
Reyhaneh Sadegh	Zadeh						
Maria	Khan						
William	Sherwood						
Claudio	Addari						
Roshni	Manex						
Nicole	Whitehead						
Fred	Wilson						
Luke	Edwards						
Kshiteeja	Nalk						
Sophie	Biddle						
Suzannah	Lant						
Francesca	Holden						
Shree	Voralia						
Nicola	Ocean						
Arun	Sahni						
Prakhar	Srilastava						
Sultan	Iqbal						
Shamil	Tana						
Vishal Venkat	Raman						

***First Name and Middle Initial(s)**	***Last Name**	***Suffix (eg, Jr, III)**	Academic Degrees	Institution	Location (city, state/province, country)	Role or Contribution, eg, chair, principal investigator	Group (if more than 1 Group listed in the byline) and/or Subgroup (eg, Steering Committee)
Zoszka	Webb						
Luke	Parker						
Arnold	Dela Rosa						
Miran	Kadr						
Eleanor	Harvey						
Ryan	Howle						
Aatif	Husain						
Olivia	Morley						
Sarah	Loftus						
Jenna	Hutchinson						
Shaman	Jhanji						
Ethel	Black						
David	Parkinson						
Ravishankar	Raobaikady						
Mark	Borthwick						
Christie	James						
Grace	Polley						
Neil	Davidson						
Sally	Beer						
Paula	Hutton						
Archana	Bashyal						
Jean	Wilson						
Soyamol	Mathew						
Jung	Ryu						
Jason	Cupitt						
Gareth	Hardy						
Leonie	Benham						
Robert	Downes						
Neil	Flint						
Michael	Little						
Ravindra	Pochiraju						

***First Name and Middle Initial(s)**	***Last Name**	***Suffix (eg, Jr, III)**	Academic Degrees	Institution	Location (city, state/province, country)	Role or Contribution, eg, chair, principal investigator	Group (if more than 1 Group listed in the byline) and/or Subgroup (eg, Steering Committee)
Prematie	Andreou						
Dawn	Hales						
Jessica	Hailstone						
Megha	Mathews						
Martin	Huntley						
Lorraine	Stephenson						
Jacqui	Hussey						
Hao-Ern	Tan						
Simon	Holbrook						
Hayley	Kemp						
David	Earl						
Richard	Innes						
Benjamin	Plumb						
Patricia	Doble						
Rebecca	Purnell						
Ashly	Thomas						
Muhammad Hamza	Noor						
Waqas	Khaliq						
Micheal	Jennings						
Bernd Oliver	Rose						
Rosaleeta	Reece-Anthony						
Sagira	Khatun						
Samantha	Dickinson						
Jayson	Clarke						
Charlie	Cox						
Adam	Longley						
Tariq	Ali						
Babita	Gurung						
Mohamed	Moubarak						
Alan	Williams						
Jonathan	Ball						

*First Name and Middle Initial(s)	*Last Name	*Suffix (eg, Jr, III)	Academic Degrees	Institution	Location (city, state/province, country)	Role or Contribution, eg, chair, principal investigator	Group (if more than 1 Group listed in the byline) and/or Subgroup (eg, Steering Committee)
Susannah	Leaver						
Sarah	Farnell-Ward						
Maria	Thanasi						
Shreeja	Dangol						
Vince	Ventura						
Massimiliano	Valcher						
Christine	Sicat						
Nikki	Yun						
Rebecca	Kanu						
Maria Maiz	Cordoba						
Ha	Trinh						
Karen	Lloyd						
Romina Pepermans	Saluzzio						
Lijun	Ding						
Helen	Farrah						
Edna	Fernandes						
Chris	Nutt						
Jon	Silversides						
Danny	McAuley						
Peter	McGuigan						
Emmet	Major						
Elliott	Lonsdale						
Nerielle	Fundano						
Kathryn	Ward						
Christine	Turley						
Aisling	O'Neill						
Stephanie	Finn						
Jackie	Green						
Erin	Collins						
Julie	McAuley						
Jeanette	Mills						

*First Name and Middle Initial(s)	*Last Name	*Suffix (eg, Jr, III)	Academic Degrees	Institution	Location (city, state/province, country)	Role or Contribution, eg, chair, principal investigator	Group (if more than 1 Group listed in the byline) and/or Subgroup (eg, Steering Committee)
Chris	Wright						
Michelle	Growcott						
Iain	McCullagh						
Stephen	Wright						
Ian	Clement						
Jonathan	Shelton						
Matthew	Faulds						
Thomas	Hellyer						
Harriet	Morton						
Christopher	Pollard						
Christopher	White						
Leigh	Dunn						
Verity	Calder						
Susan	Taylor						
Pamela	Garcia						
Benjamin	Brown						
James	Savage						
Maite	Babio-Galan						
Kimberley	Webster						
Tessa	Wilkinson						
Arti	Gulati						
Tara	Shrestha						
Carole	Hays						
Lauren	Butler						
Fatima	Simoes						
Margaret	McNeil						
Ian	Storey						
Simon	Whiteley						
Elizabeth	Wilby						
Susan	Trott						
Sarah	Watts						

***First Name and Middle Initial(s)**	***Last Name**	***Suffix (eg, Jr, III)**	Academic Degrees	Institution	Location (city, state/province, country)	Role or Contribution, eg, chair, principal investigator	Group (if more than 1 Group listed in the byline) and/or Subgroup (eg, Steering Committee)
Shailamma	Mathew						
Sheila	Salada						
Adam	Neep						
Nora	Youngs						
Clare	Howcroft						
Matthew	Powell						
Michael	Adlam						
Elankumaran Paramasivam							
Zoe	Friar						
David	Antcliffe						
Stephen	Brett						
Anthony	Gordon						
Dorota	Banach						
Roceld	Rojo						
Sonia Sousa	Arias						
Ziortza	Fernandez de Pinedo Artaraz						
Phoebe	Coghlan						
Amal	Mohammed						
Eleanor	Jepson						
Jenny	Wong						
Anita Tamang	Gurung						
Caoimhe	O’Dwyer						
Sara	Perez Guillotin						
Maie	Templeton						
James	Hanison						
Jonathan	Bannard-Smith						
Daniel	Conway						
Shoneen	Abbas						
Mohamad	Aly						
Stephen	Benington						
Teh	Eng Hean						

***First Name and Middle Initial(s)**	***Last Name**	***Suffix (eg, Jr, III)**	Academic Degrees	Institution	Location (city, state/province, country)	Role or Contribution, eg, chair, principal investigator	Group (if more than 1 Group listed in the byline) and/or Subgroup (eg, Steering Committee)
Daniel	Hayley						
Ellen	McGuckin						
Andrew	Martin						
Thomas	Morris						
William	Musselbrook						
Bhaskar	Narayan						
Thomas	Wright						
Chris	Wheeler						
Melanie	Barker						
Richard	Clark						
Emma	Connaughton						
Rose	Jama						
Deborah	Paripoorani						
Rachael	Quayle						
Anila	Sukumaran						
Charlotte	Taylor						
Megan	Balmer						
Saejohn	Lingeswaran						
Lauren	Edmunds						
Katharine	Wylie						
Andrew	Owen						
Gavin	Perkins						
Sean	Munnelly						
Daniel	Park						
Jo	Gresty						
Ellie	Reeves						
Celina	Maliaykal						
Teresa	Melody						
Jacobus	Preller						
Petra	Polgarova						
Cristina	Bravoelvira						

***First Name and Middle Initial(s)**	***Last Name**	***Suffix (eg, Jr, III)**	Academic Degrees	Institution	Location (city, state/province, country)	Role or Contribution, eg, chair, principal investigator	Group (if more than 1 Group listed in the byline) and/or Subgroup (eg, Steering Committee)
Sofia	Teixeira						
James	Varley						
Sapna Sharma	Hajela						
Kay	Elston						
Siobhan	Campbell						
Meike	Keil						
Muhammad	Elbehery						
Jocelyn	Marshall						
Susan	Stevenson						
Andrew	Conway Morris						
Prasad	Gogineni Venkateskara						
Michael	Reay						
Karen	Reid						
Rebecca	Brown						
Chinenyenwa	Amareihe						
Elliot	Yates						
Jia	Luen Goh						
Edward	Jones						
Aamer	Mughal						
David	Brealey						
Niall	MacCallum						
Samuel	Clark						
Deborah	Smyth						
Georgia	Bercades						
Ingrid	Hass						
Gladys	Martir						
Jung	Ryu						
Anna	Reyes						
Maria Alexandra Za	Martinez						
Laura	Gallagher						
Chi	Yee Chung						

***First Name and Middle Initial(s)**	***Last Name**	***Suffix (eg, Jr, III)**	Academic Degrees	Institution	Location (city, state/province, country)	Role or Contribution, eg, chair, principal investigator	Group (if more than 1 Group listed in the byline) and/or Subgroup (eg, Steering Committee)
Graeme	Sanders						
Vipal	Chawla						
Namrata	Maheshwari						
Tessa	Glazebrook						
Hollie	Angel						
Rebecca	Squires						
Hayley	Dolan						
Christopher	Donnelly						
Lucy	Mires						
Robert	Musalagani						
Suzanne	Williams						
Robin	Heij						
Peter	Young						
Mark	Blunt						
Gayathri	Wijewardena						
John	Gibson						
Aricsa Mariya	Joshy						
Jeremy	Bewley						
Kieron	Rooney						
Katie	Sweet						
Kim	Wright						
Lisa	Grimmer						
Denise	Webster						
Casandra	Bazan Lacerot						
Rachel	Shiel						
Eva Maria	Hernandez Morano						
Christina	Coleman						
Eleanor	Daniel						
Oluwatosin	Komolafe						
Josephine	Bonnici						
Linda	Pipira						

***First Name and Middle Initial(s)**	***Last Name**	***Suffix (eg, Jr, III)**	Academic Degrees	Institution	Location (city, state/province, country)	Role or Contribution, eg, chair, principal investigator	Group (if more than 1 Group listed in the byline) and/or Subgroup (eg, Steering Committee)
Rebekah	Johnson						
Anna	Chillingworth						
Ya-Hui	Liang						
Georgia	Efford						
Angeliki	Kolovou						
George	Davies						
Zoe	Garland						
Bethany	Gumbrill						
Ivan	Collin						
Matthew	Gibbins						
Thomas	Brougham						
Agnieszka	Skorko						
Dan	Harvey						
William	Phipps						
Kathryn	Harrold						
Nick	Plummer						
Ben	Lowe						
Paul	James						
Sara	Ahmed						
Rukmini	Ghosh						
Omer	Mohamed						
Tanushree	Santra						
James	Shilston						
Andrew	Russell						
Viresh	Patel						
Upasana	Topiwala						
Habideen	Bello						
Julia	Sampson						
Lucy	Ryan						
Cecilia	Peters						
Megan	Meredith						

*First Name and Middle Initial(s)	*Last Name	*Suffix (eg, Jr, III)	Academic Degrees	Institution	Location (city, state/province, country)	Role or Contribution, eg, chair, principal investigator	Group (if more than 1 Group listed in the byline) and/or Subgroup (eg, Steering Committee)
Louise	Conner(Now Hughes)						
Lucy	Morris						
Amy	Clark						
Alice	Baddeley						
Lisa	Mcloughlin						
Cate	Walton						
Treesa	Joseph						
Anju	Thomas						
Sophie	Lubbock						
David	Ford						
Alexandra	McCoy						
Tony	N'Dungu						
Ingeborg	Welters						
Vinoth	Sankar						
Alicia	Waite						
Brian	Johnston						
David	Shaw						
Vicki	Waugh						
Karen	Williams						
Maria	Lopez Martinez						
Maria	Norris						
Maria Arra	Carlota Mahiya						
Jamie	Fernandez Roman						
Jin-Xi.	Yuan						
Silvia	Manes						
Caitlin	Lythgoe						
Ibrahim	Almafreji						
Josh	Colfar						
Laura	Medhurst						
Stephanie	Beresford						
Sofia	Farina						

*First Name and Middle Initial(s)	*Last Name	*Suffix (eg, Jr, III)	Academic Degrees	Institution	Location (city, state/province, country)	Role or Contribution, eg, chair, principal investigator	Group (if more than 1 Group listed in the byline) and/or Subgroup (eg, Steering Committee)
Lema	Imam						
Syamlam	Ali						
Zachary	Thomas						
Francesca	Bold						
Edward	Hughes						
Katherine	Hodson						
Aleem	Morenikeji						
Daniel	Watkin						
Tamas	Szakmany						
Amy	Cardwell						
Anne	Frawley						
Marlies	Ostermann						
Gillian	Radcliffe						
Nicholas	Barrett						
Simon	Sparkes						
Adam	Woodman-Bailey						
Eirini	Kosifidou						
Aneta	Bociek						
Ellie	Hendrie						
Rosario	Lim						
Fabiola	D’Amato						
Sarah	Fordyce						
Benjie	Cendreda						
Kyma	Morera Vas						
Jacqueline	Pan						
Christopher	Meddings						
Vladimir	Milic						
Mike	Barker						
Jennifer	Owusu-Afriyie						
Carolin	Engelhard						
Malcolm	Sim						

*First Name and Middle Initial(s)	*Last Name	*Suffix (eg, Jr, III)	Academic Degrees	Institution	Location (city, state/province, country)	Role or Contribution, eg, chair, principal investigator	Group (if more than 1 Group listed in the byline) and/or Subgroup (eg, Steering Committee)
Richard	Appleton						
Maximilian	Ralston						
Andrew	Arnott						
Steven	Henderson						
Izabela	Orlikowska						
Sophie	Kennedy-Hay						
Christopher	Murray						
Matthew	Devine						
Padraig	Headley						
John	McCaffrey						
Daniel	Donnelly						
Richard	Young						
Samantha	Hagan						
Victoria	Adell						
Elizabeth	Murphy						
Alasdair	Hay						
Jian	Quek						
Stephen	Wilson						
Catherine	Jardine						
Mark	Forrest						
Emma	Collins						
Miqdad	Ibrahim						
Mark	Wheeley						
Mostafa	Kodous						
Mathew	Blake						
Victoria	Lacey						
Michael	Eager						
Robin	Jootun						
Janine	Birch						
