# Supplementary material for: Cost-Effectiveness of α2 Agonists for Intravenous Sedation in Patients With Critical Illness
Source: JAMA Netw Open. 2025 May 19;8(5):e2517533. doi: 10.1001/jamanetworkopen.2025.17533 (PMC12090026; doi:10.1001/jamanetworkopen.2025.17533)
Supplement: Supplement 3. — Data Sharing Statement [file jamanetwopen-e2517533-s003.pdf]

Data Sharing Statement

Morris. Cost-Effectiveness of α_2 Agonists for Intravenous Sedation in Patients With Critical Illness. *JAMA Netw Open*. Published online May 19, 2025. doi:10.1001/jamanetworkopen.2025.17533

Data

Data available: Yes
Data types: Deidentified participant data
How to access data: De-identified participant data may be made available after publication on request by application to the corresponding author and after assenting to a data access agreement.
When available: With publication

Supporting Documents

Document types: None

Additional Information

Who can access the data: Researchers whose proposed use of the data has been approved.
Types of analyses: Economic analyses.
Mechanisms of data availability: Without investigator support, after approval of a proposal, with a signed data access agreement.
